# Supplementary material for: The Hydractinia cell atlas reveals cellular and molecular principles of cnidarian coloniality
Source: Nat Commun. 2025 Mar 3;16:2121. doi: 10.1038/s41467-025-57168-z (PMC11876637; doi:10.1038/s41467-025-57168-z)
Supplement: Supplementary file 1 — Supplementary Information [file 41467_2025_57168_MOESM1_ESM.pdf]

Supplementary Information

The *Hydractinia* cell atlas reveals cellular and molecular principles of cnidarian coloniality

David A. Salamanca-Díaz [1, 2, 3, †], Helen R. Horkan [4, 5, †, *], Helena García-Castro [1, 2, 3], Elena Emili [1,6], Miguel Salinas-Saavedra [4], Alberto Pérez-Posada [1, 2, 3], Maria Eleonora Rossi [4,7], Marta Álvarez-Presas [7,8], Rowan Mac Gabhann [4], Paula Hillenbrand [4], Febrimarsa [4,9], Camille Curantz [4,10], Paris K. Weavers [4], Yasmine Lund-Ricard [4], Tassilo Förg[11], Manuel H. Michaca[12], Steven M. Sanders[12], Nathan J. Kenny [13], Jordi Paps [7], Uri Frank [4, *], Jordi Solana [1, 2, 3 *]

[1] Department of Biological and Medical Sciences, Oxford Brookes University, Oxford, UK

[2] Living Systems Institute, University of Exeter, Exeter, UK

[3] Department of Biosciences, University of Exeter, Exeter, UK

[4] Centre for Chromosome Biology, School of Biological and Chemical Sciences, University of Galway, Galway, Ireland

[5] Present address: Stowers Institute for Medical Research, Kansas City, MO, USA

[6] Present address: Department of Systems Medicine, University of Rome "Tor Vergata", 00133 Rome, Italy

[7] School of Biological Sciences, University of Bristol, Bristol, UK

[8] Present address: Institut de Biologia Evolutiva (CSIC-Universitat Pompeu Fabra), Passeig Marítim de la Barceloneta, Barcelona, Spain

[9] Present address: Faculty of Pharmacy, Universitas Muhammadiyah Surakarta, Jawa Tengah, Indonesia

[10] Present address: Sorbonne Université, Institut de Biologie Paris-Seine (IBPS), Paris, France

[11] Institute of Zoology, University of Heidelberg, Heidelberg, Germany

[12] Thomas E. Starzl Transplantation Institute, University of Pittsburgh, PA, USA.

[13] Department of Biochemistry, University of Otago, P.O. Box 56, Dunedin, Aotearoa New Zealand

[*] Corresponding authors

[†] Equally contributing authors

Contents:

Supplementary Note 1

Supplementary Figure 1

Supplementary Figure 2

Supplementary Figure 3

Supplementary Figure 4

Supplementary Figure 5

Supplementary Figure 6

Supplementary Figure 7

Supplementary References

Supplementary Note 1: Explanation of cluster naming and grouping.

i-cells, early and mixed progenitors (Clusters 4, 8, 14): We identified i-cells and their early progeny (Clusters 14 and 4, respectively) based on high *Piwi1* (LOC130623353) and *Piwi2* (LOC130628528) expression and an absence or low levels of cell type specific marker expression, respectively, indicating an undifferentiated cell state. Cluster 14 contains 5.6% of the total number of cells captured from the feeding polyp only sub-libraries, while in the animal, true pluripotent i-cells represent 1% or less of the total cell population of feeding polyps, as verified by flow cytometry and single-cell transplantation of transgenic reporter animals[1, 2, 3], *Piwi1* and *Piwi2* were also expressed in cluster 8 but this cluster exhibits markers of specific cell types, as well as epigenetic regulators.

sperm and spermatogenesis (Clusters 6, 34): Clusters 6 and 34 were determined to be sperm and spermatogenesis due to expression of *Piwi1* and *Piwi2*, and expression of spermatogenesis specific markers including *Histone H2b.3/4* [4], sperm specific antigen 16 (LOC130622507), *Histone H2b.6* (LOC130636493), *Tubulin alpha 1 chain* (LOC130641471), and *Myosin 4-like* (LOC130621975).

Neurones (Clusters 16, 26, 27, 29, 33): This group of clusters was identified as neuronal due to expression of ELAV orthologs (*Elav1*; LOC130630563, *Elav2*; LOC130612043 and*Elav3*; LOC130630562) [5, 6]. Cluster 16 was further identified as *RFamide* and *GLWamide* neurones (LOC130657354, LOC130624206 respectively)[2], Clusters 26 and 33 were identified as *PQRFVamide* neurones (LOC130628716) and Cluster 26 was further named *Involucrin*+ neurones (LOC130654945). Cluster 27 was identified as *Fax*+ neurones (LOC130645007) and Cluster 29 was identified as *Pkdrej*+ neurones (LOC130623103). Additionally, all of these clusters co-occur in Supplementary Figure 1F.

Nematoblasts and nematocytes (Clusters 10, 12, 15, 18, 25): We identified Cluster 12 as nematoblasts 1 'nb 1' due to expression of *Piwi1*, *Piwi2* and *SoxB2* and shared expression of marker genes with cluster 25. Clusters 12, 18 and 25 express *Ncol1* (LOC130622551) a nematogenesis marker[7]. We named Cluster 25 'nb 3+ nematoblast' because even though the expression of *HsymJFT1c-I* (LOC130642394) predominates in this cluster, it shares many markers with other nematoblast clusters. Cluster 18 '*Hrnr*+ nematoblast' expresses *HsymJFT2a* and *Hornerin-like* orthologs (LOC130641429 and LOC130653621, respectively). Additionally, Clusters 12, 18 and 25 co-occur in Supplementary Figure 1F. Clusters 10 and 15 were showing expression of *Trichohyalin-like* orthologs and *ARSTNd2*[8] (LOC130657211, LOC130662747 and LOC130644310 respectively), and do not co-occur with any other cluster. We classify them as terminally differentiated nematocytes and name them '*Tchh1* & *2* + nematocytes'.

***Conodipine*+ cells (Clusters 9, 13, 36):** Cluster 9 and 36 are labelled '*Conodipine*+ cells' as they share markers with epithelial cells but have specific expression of *Conodipine* orthologs (LOC130644743, LOC130636858, LOC130642091). Also, they express at least one homolog of the Membrane Attack Complex and perforin family (MAC), a group of proteins found in cnidarians (LOC130635919), expressed in venom cells, so we classify these cells as venomous epithelial cells[9]. While neither of these clusters co-occur with any particular other in Supplementary Figure 1F, Cluster 13 groups with Cluster 9 and is likely to be a *Conodipine*+ cell type.

Gland (Clusters 17, 22, 24, 21, 32): Digestive gland cells include lusters 17, 22, 24) Cluster 17 was identified as a gland cell type due to strong expression of known gland cell markers *Astacin1* (LOC130623614), *Astacin2* (LOC130614501)[10], along with lower expression of *Chitinase1, 2* and *3* (LOC130649211, LOC130624325, LOC130655910 respectively) and a strong expression of zinc metalloproteinase *Nas 4-like* (LOC130614209). Cluster 22 reveals a strong expression of zinc

metalloproteinase *Nas14-like* orthologs (LOC130636648, LOC130614442), together with expression of *Astacin 1* and *2*, at a lower level than in cluster 17. Cluster 24, *Chitinase2*+ cells, had weak expression of *Astacin 1* and *2* but strong expression of *Chitinase 1, 2* and *3* along with specific expression of *Antistasin* orthologs (LOC130646107,LOC130657200, LOC130646090). Clusters 17, 22 and 24 co-occur and are likely to be digestive gland cell types (Supplementary Figure 1F).Mucosal gland cells inlcude Clusters 21, 32. *Rsp*+ cells, Cluster 21, were named after the specific ortholog markers of rhamnospondin (LOC130621200, LOC130655212, LOC130622532), a known gland cell marker likely for production of the outer mucosal layer (glycocalyx)[11]. Cluster 32 was identified as gland 4 cells due to the shared low expression of one rhamnospondin ortholog (LOC130622532). Due to co-occurrence of Clusters 21, 32 (Supplementary Figure 1F), we classify these cells as mucosal gland cells based on their secretory nature.

Epithelial (Clusters 0, 1, 7, 19, 23, 28, 30, 35, 37): Clusters 0, 1, 7, 19, 23, 28, 30, 35, 37 were labelled as epithelial due to expression of general epithelial cell markers like proton-coupled zinc antiporter *SLC30A1*, *Protocadherin Fat 4* orthologs and *Alpha catulin-like* (LOC130626173, LOC130648302, LOC130647734, LOC130655582 and LOC130641787 respectively) [PMID: 18574100] and an absence of known muscle markers. Many markers of these clusters also closely co-occur with the epitheliomuscular group (Supplementary Figure 1F).

Epitheliomuscular (Clusters 2, 3, 5, 20, 31): Clusters 2, 3, 5, 20, and 31 were identified as epitheliomuscular cells due to expression of known muscle marker genes *Myosin heavy chain* (striated muscle-like) (LOC130649617), *Tropomyosin* (LOC130645893) [12], *Myophilin* orthologs (LOC130614185, LOC130613823, LOC130644608), *Titin-like* (LOC120641779) [PMID: 20018670, PMID: 28168188, PMID: 8740429], and close co-occurrence (Supplementary Figure 1F). This group also broadly co-occurs with the epithelial group and as such is a subclassification of epithelial cells. Clusters 2, 3, 31 are enriched in feeding polyps only and have specific expression of *Rhamnose-binding lectin-like* (LOC130635948) which may be specific to the mouth of feeding polyps[13].

***Prisilkin-like*+ cells (Cluster 11):** Cluster 11 was highly enriched in the stolon specific sample, but also contains cells from the mixed polyp type sample (likely due to the gradual transition between polyp and stolon). Cluster 11 co-occurs with epithelial cells but has specific expression of *Prisilkin* (LOC130630016), which is involved in mantle production in oyster and sea snail[14] and binds tightly to chitin.

Unannotated (Clusters 37, 38, 39, 40, 41, 42, 43, 44, 45, 46, 47, 48, 49, 50, 51, 52): These clusters were left unannotated either due to a lack of specific marker genes, a lack of annotated marker genes or lack of correlation with known clusters in the co-occurrence analysis. Additionally, these clusters appear to be small and distributed sporadically in the UMAP space, often with cells in multiple larger clusters. As such we believe these clusters are largely composed of artefacts such as doublets and cells with low read counts.

Supplementary Figure 1.

A: Violin plots of the number of genes and counts across all libraries. B: Violin plot showing the number of genes per cluster. C: Violin plot showing the number of counts per cluster. D-E: Violin plots showing number of genes and counts per colony part. F: Heatmap of the co-occurrence matrix showing similarities between cell types based on gene expression correlation.

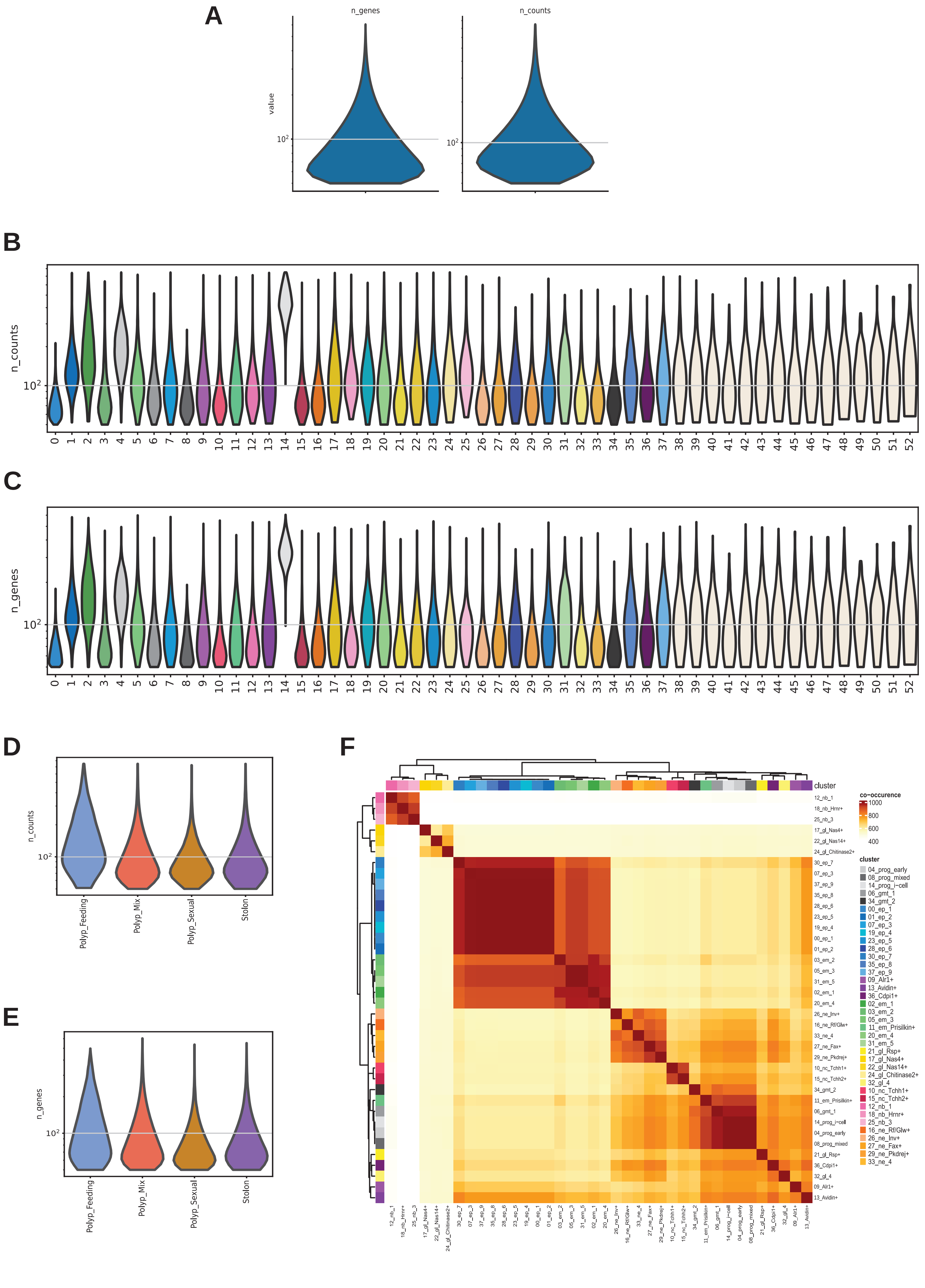

A
n_genes
n_counts
value
10^2
B
n_counts
10^2
0 1 2 3 4 5 6 7 8 9 10 11 12 13 14 15 16 17 18 19 20 21 22 23 24 25 26 27 28 29 30 31 32 33 34 35 36 37 38 39 40 41 42 43 44 45 46 47 48 49 50 51 52
C
n_genes
10^2
0 1 2 3 4 5 6 7 8 9 10 11 12 13 14 15 16 17 18 19 20 21 22 23 24 25 26 27 28 29 30 31 32 33 34 35 36 37 38 39 40 41 42 43 44 45 46 47 48 49 50 51 52
D
n_counts
10^2
Polyp_Feeding
Polyp_Mix
Polyp_Sexual
Stolon
E
n_genes
10^2
Polyp_Feeding
Polyp_Mix
Polyp_Sexual
Stolon
F
cluster
co-occurence
1000
800
600
400
cluster
04_prog_early
08_prog_mixed
14_prog_i-cell
06_gmt_1
34_gmt_2
00_ep_1
01_ep_2
07_ep_3
19_ep_4
23_ep_5
28_ep_6
30_ep_7
35_ep_8
37_ep_9
09_Alr1+
13_Avidin+
36_Cdpi1+
02_em_1
03_em_2
05_em_3
11_em_Prisilkin+
20_em_4
31_em_5
21_gl_Rsp+
17_gl_Nas4+
22_gl_Nas14+
24_gl_Chitinase2+
32_gl_4
10_nc_Tchh1+
15_nc_Tchh2+
12_nb_1
18_nb_Hrnr+
25_nb_3
16_ne_Rf/Glw+
26_ne_Inv+
27_ne_Fax+
29_ne_Pkdrej+
33_ne_4
12_nb_1
18_nb_Hrnr+
25_nb_3
17_gl_Nas4+
22_gl_Nas14+
24_gl_Chitinase2+
30_ep_7
07_ep_3
37_ep_9
35_ep_8
28_ep_6
23_ep_5
19_ep_4
00_ep_1
01_ep_2
03_em_2
05_em_3
31_em_5
02_em_1
20_em_4
26_ne_Inv+
16_ne_Rf/Glw+
33_ne_4
27_ne_Fax+
29_ne_Pkdrej+
10_nc_Tchh1+
15_nc_Tchh2+
34_gmt_2
11_em_Prisilkin+
06_gmt_1
14_prog_i-cell
04_prog_early
08_prog_mixed
21_gl_Rsp+
36_Cdpi1+
32_gl_4
09_Alr1+
13_Avidin+

Supplementary Figure 2.

A. Percentage of cells for each resolution belonging to the corresponding reanalysed subset. Cells in each subset resolution have been labelled transferring the identities from the clustering annotation in the whole dataset. From here, the colours of the clusters have been assigned according to the label most represented. B. UMAPs from each subset with the different resolutions. C. UMAPs from each subset with annotation transfer from the whole original dataset.

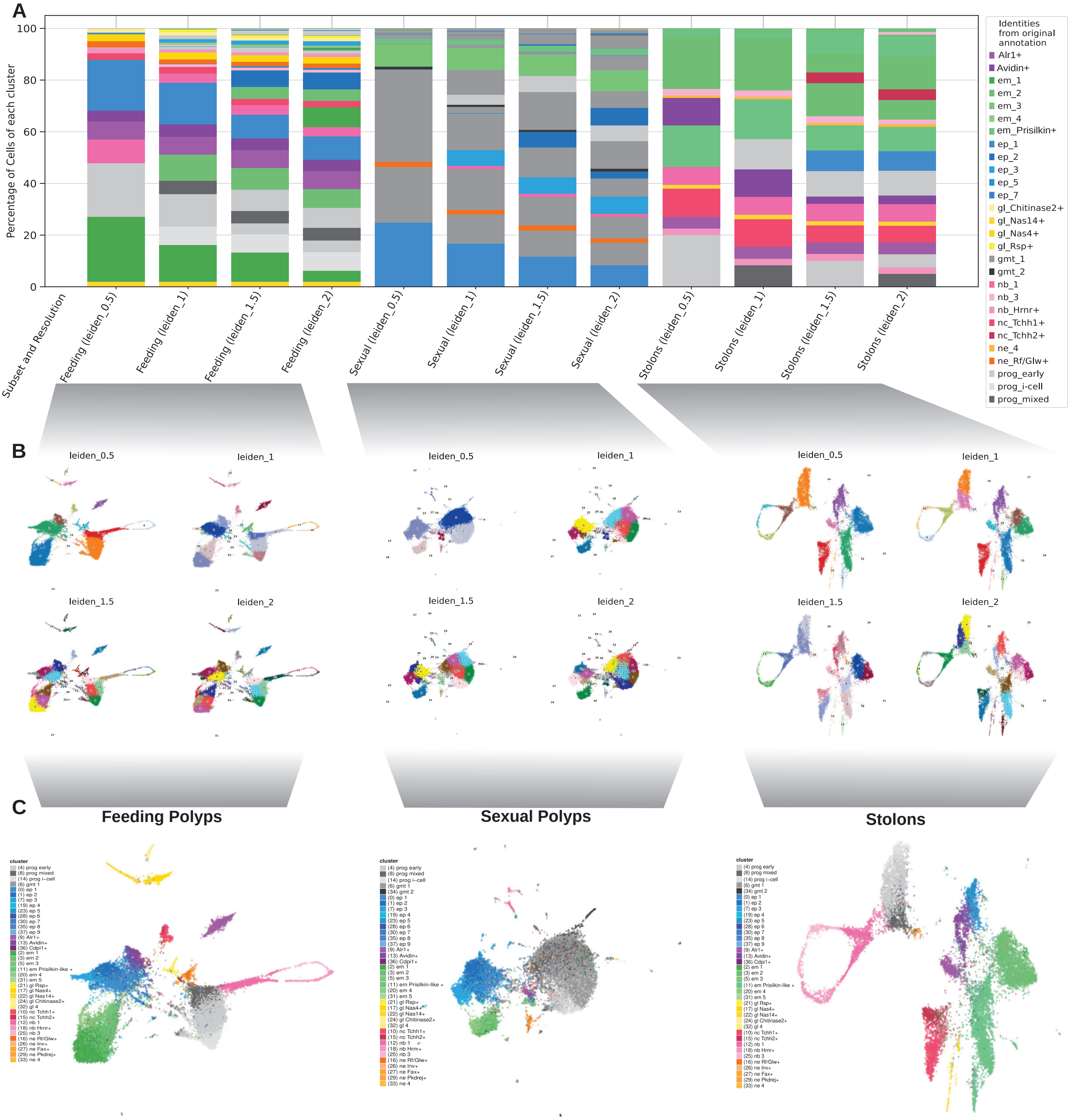
A
Percentage of Cells of each cluster
100
80
60
40
20
0
Subset and Resolution
Feeding (leiden_0.5)
Feeding (leiden_1)
Feeding (leiden_1.5)
Feeding (leiden_2)
Sexual (leiden_0.5)
Sexual (leiden_1)
Sexual (leiden_1.5)
Sexual (leiden_2)
Stolons (leiden_0.5)
Stolons (leiden_1)
Stolons (leiden_1.5)
Stolons (leiden_2)
Identities from original annotation
Alr1+
Avidin+
em_1
em_2
em_3
em_4
em_Prisilkin+
ep_1
ep_2
ep_3
ep_5
ep_7
gl_Chitinase2+
gl_Nas14+
gl_Nas4+
gl_Rsp+
gmt_1
gmt_2
nb_1
nb_3
nb_Hrnr+
nc_Tchh1+
nc_Tchh2+
ne_4
ne_Rf/Glw+
prog_early
prog_i-cell
prog_mixed
B
leiden_0.5
leiden_1
leiden_1.5
leiden_2
C
Feeding Polyps
Sexual Polyps
Stolons
cluster

Supplementary Figure 3.

A: Barplot showing number of genes quantified with more than 5 UMI counts per million (cpm) per cluster in a pseudobulk analysis. B: Boxplot of the log transformation of the gene counts per cluster showing the distribution of the data.

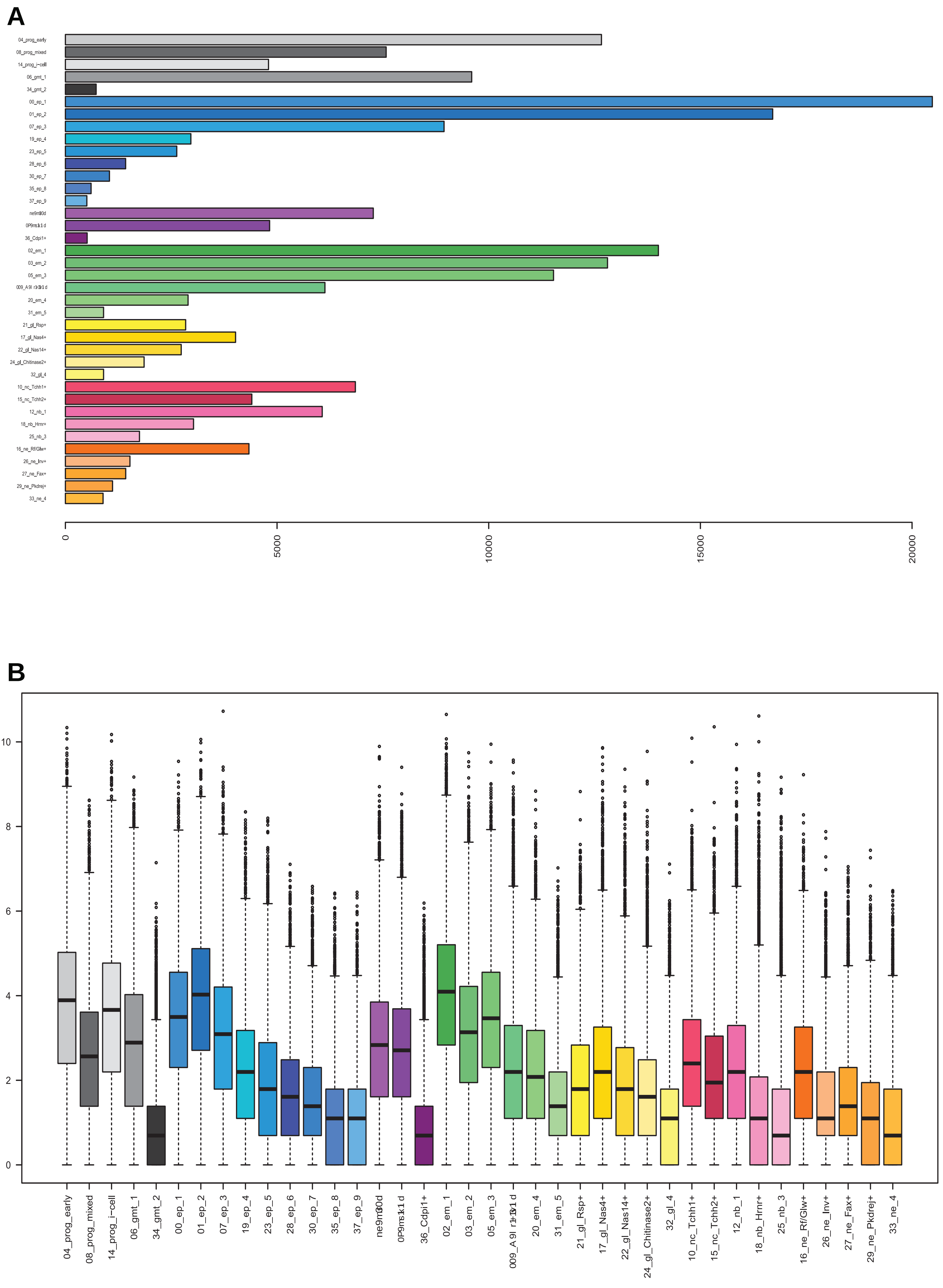
A
B
04_prog_early
08_prog_mixed
14_prog_i−cell
06_gmt_1
34_gmt_2
00_ep_1
01_ep_2
07_ep_3
19_ep_4
23_ep_5
28_ep_6
30_ep_7
35_ep_8
37_ep_9
36_Cdpi1+
02_em_1
03_em_2
05_em_3
20_em_4
31_em_5
21_gl_Rsp+
17_gl_Nas4+
22_gl_Nas14+
24_gl_Chitinase2+
32_gl_4
10_nc_Tchh1+
15_nc_Tchh2+
12_nb_1
18_nb_Hrnr+
25_nb_3
16_ne_Rf/Glw+
26_ne_Inv+
27_ne_Fax+
29_ne_Pkdrej+
33_ne_4
0
5000
10000
15000
20000
0
2
4
6
8
10

Supplementary Figure 4.

Bayesian phylogenetic tree of full length POU protein sequences.

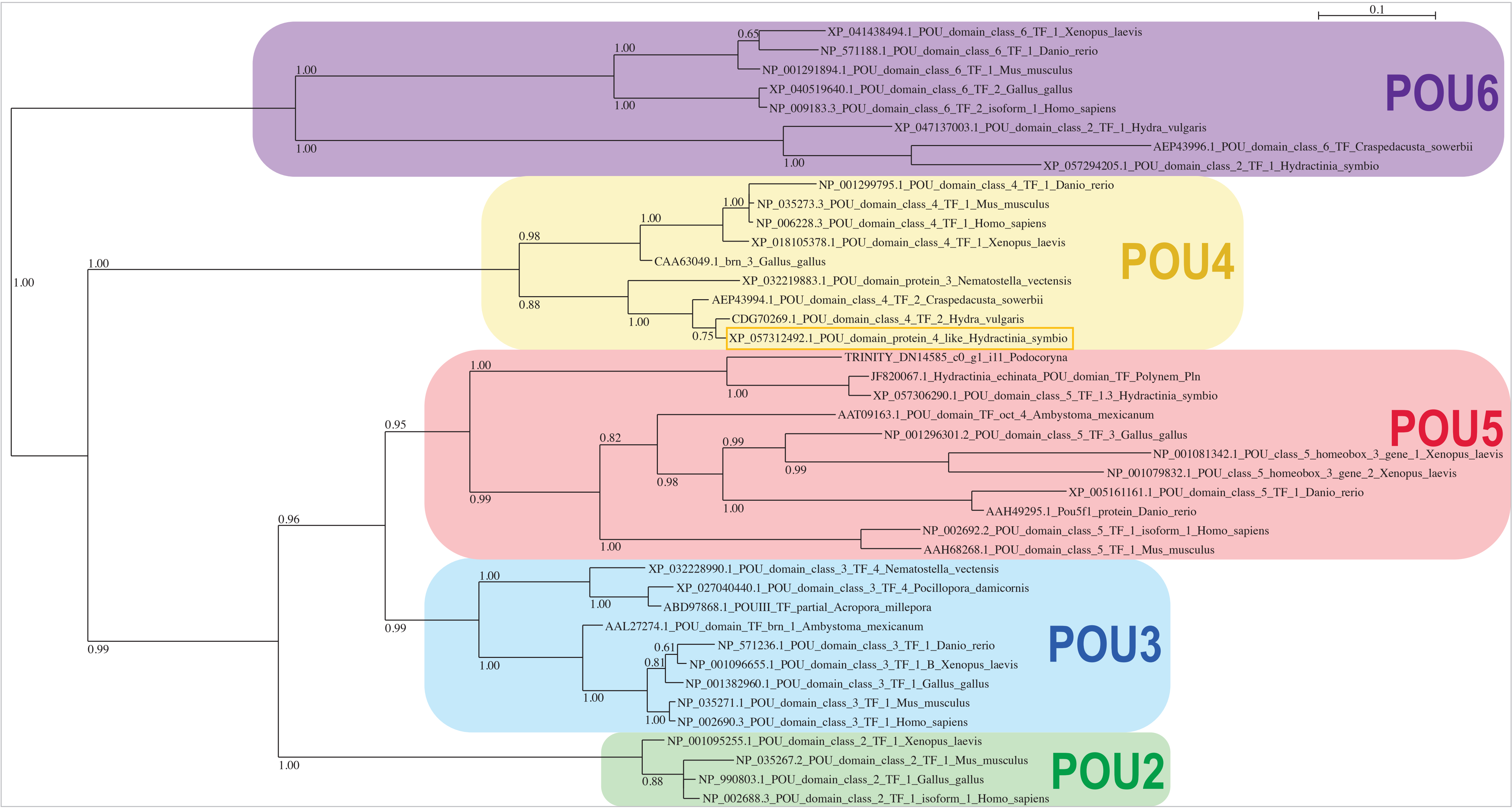
0.1
XP_041438494.1_POU_domain_class_6_TF_1_Xenopus_laevis
NP_571188.1_POU_domain_class_6_TF_1_Danio_rerio
NP_001291894.1_POU_domain_class_6_TF_1_Mus_musculus
XP_040519640.1_POU_domain_class_6_TF_2_Gallus_gallus
NP_009183.3_POU_domain_class_6_TF_2_isoform_1_Homo_sapiens
XP_047137003.1_POU_domain_class_2_TF_1_Hydra_vulgaris
AEP43996.1_POU_domain_class_6_TF_Craspedacusta_sowerbii
XP_057294205.1_POU_domain_class_2_TF_1_Hydractinia_symbio
POU6
NP_001299795.1_POU_domain_class_4_TF_1_Danio_rerio
NP_035273.3_POU_domain_class_4_TF_1_Mus_musculus
NP_006228.3_POU_domain_class_4_TF_1_Homo_sapiens
XP_018105378.1_POU_domain_class_4_TF_1_Xenopus_laevis
CAA63049.1_brn_3_Gallus_gallus
XP_032219883.1_POU_domain_protein_3_Nematostella_vectensis
AEP43994.1_POU_domain_class_4_TF_2_Craspedacusta_sowerbii
CDG70269.1_POU_domain_class_4_TF_2_Hydra_vulgaris
XP_057312492.1_POU_domain_protein_4_like_Hydractinia_symbio
POU4
TRINITY_DN14585_c0_g1_i11_Podocoryna
JF820067.1_Hydractinia_echinata_POU_domian_TF_Polynem_Pln
XP_057306290.1_POU_domain_class_5_TF_1.3_Hydractinia_symbio
AAT09163.1_POU_domain_TF_oct_4_Ambystoma_mexicanum
NP_001296301.2_POU_domain_class_5_TF_3_Gallus_gallus
NP_001081342.1_POU_class_5_homeobox_3_gene_1_Xenopus_laevis
NP_001079832.1_POU_class_5_homeobox_3_gene_2_Xenopus_laevis
XP_005161161.1_POU_domain_class_5_TF_1_Danio_rerio
AAH49295.1_Pou5f1_protein_Danio_rerio
NP_002692.2_POU_domain_class_5_TF_1_isoform_1_Homo_sapiens
AAH68268.1_POU_domain_class_5_TF_1_Mus_musculus
POU5
XP_032228990.1_POU_domain_class_3_TF_4_Nematostella_vectensis
XP_027040440.1_POU_domain_class_3_TF_4_Pocillopora_damicornis
ABD97868.1_POUIII_TF_partial_Acropora_millepora
AAL27274.1_POU_domain_TF_brn_1_Ambystoma_mexicanum
NP_571236.1_POU_domain_class_3_TF_1_Danio_rerio
NP_001096655.1_POU_domain_class_3_TF_1_B_Xenopus_laevis
NP_001382960.1_POU_domain_class_3_TF_1_Gallus_gallus
NP_035271.1_POU_domain_class_3_TF_1_Mus_musculus
NP_002690.3_POU_domain_class_3_TF_1_Homo_sapiens
POU3
NP_001095255.1_POU_domain_class_2_TF_1_Xenopus_laevis
NP_035267.2_POU_domain_class_2_TF_1_Mus_musculus
NP_990803.1_POU_domain_class_2_TF_1_Gallus_gallus
NP_002688.3_POU_domain_class_2_TF_1_isoform_1_Homo_sapiens
POU2

Supplementary Figure 5.

A: Expression plot of Alr2. B: Bubble plot of expression of all Alr and Alr-like genes highlighting in bold the expression across clusters of Alr1 and Alr2.

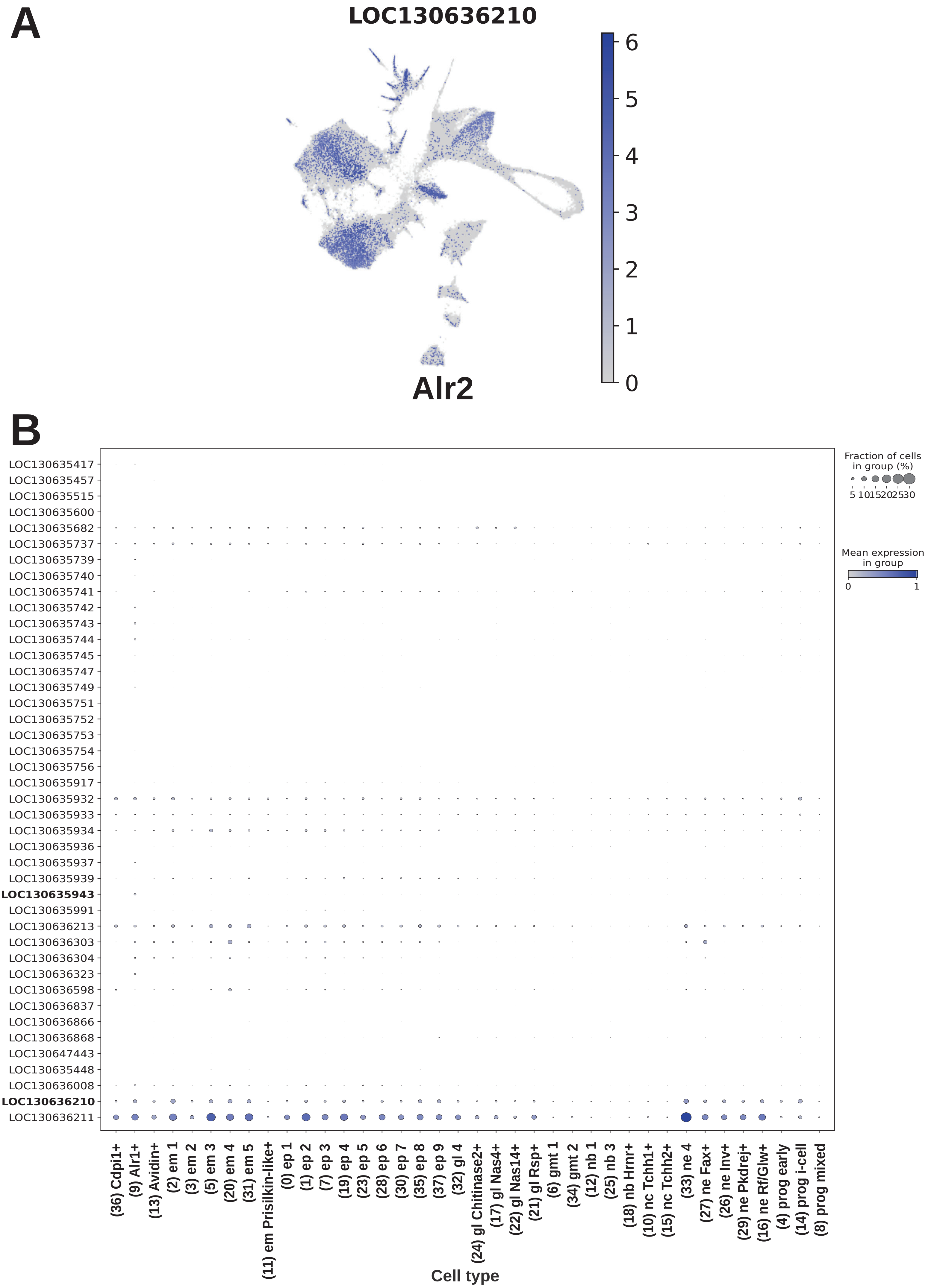
A
LOC130636210
Alr2
0
1
2
3
4
5
6
B
LOC130635417
LOC130635457
LOC130635515
LOC130635600
LOC130635682
LOC130635737
LOC130635739
LOC130635740
LOC130635741
LOC130635742
LOC130635743
LOC130635744
LOC130635745
LOC130635747
LOC130635749
LOC130635751
LOC130635752
LOC130635753
LOC130635754
LOC130635756
LOC130635917
LOC130635932
LOC130635933
LOC130635934
LOC130635936
LOC130635937
LOC130635939
LOC130635943
LOC130635991
LOC130636213
LOC130636303
LOC130636304
LOC130636323
LOC130636598
LOC130636837
LOC130636866
LOC130636868
LOC130647443
LOC130635448
LOC130636008
LOC130636210
LOC130636211
Fraction of cells in group (%)
5 10 15 20 25 30
Mean expression in group
0
1
(36) Cdpi1+
(9) Alr1+
(13) Avidin+
(2) em 1
(3) em 2
(5) em 3
(20) em 4
(31) em 5
(11) em Prisilkin-like+
(0) ep 1
(1) ep 2
(7) ep 3
(19) ep 4
(23) ep 5
(28) ep 6
(30) ep 7
(35) ep 8
(37) ep 9
(32) gl 4
(24) gl Chitinase2+
(17) gl Nas4+
(22) gl Nas14+
(21) gl Rsp+
(6) gmt 1
(34) gmt 2
(12) nb 1
(25) nb 3
(18) nb Hrnr+
(10) nc Tchh1+
(15) nc Tchh2+
(33) ne 4
(27) ne Fax+
(26) ne Inv+
(29) ne Pkdrej+
(16) ne Rf/Glw+
(4) prog early
(14) prog i-cell
(8) prog mixed
Cell type

Supplementary Figure 6.

Western blot validation of Alr1 antibody in digested and undigested conditions.

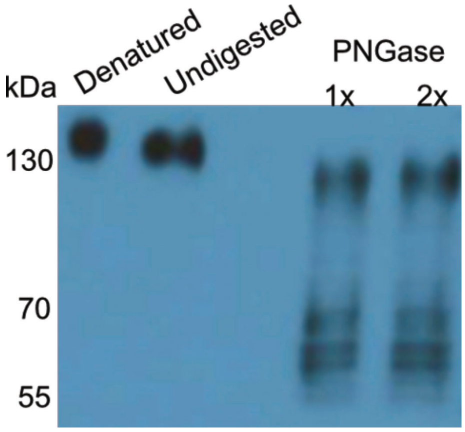
kDa
Denatured
Undigested
PNGase
1x
2x
130
70
55

Supplementary Figure 7.

Sections of Chromosome 2 and 3 from the genome assembly of *Hydractinia* showing the expression dotplots in the atlas, signal peptide regions, glycine repetitions and genomic locations of all the genes closely located or similar in sequence to *Shematrin* or *Prisilkin* orthologs.

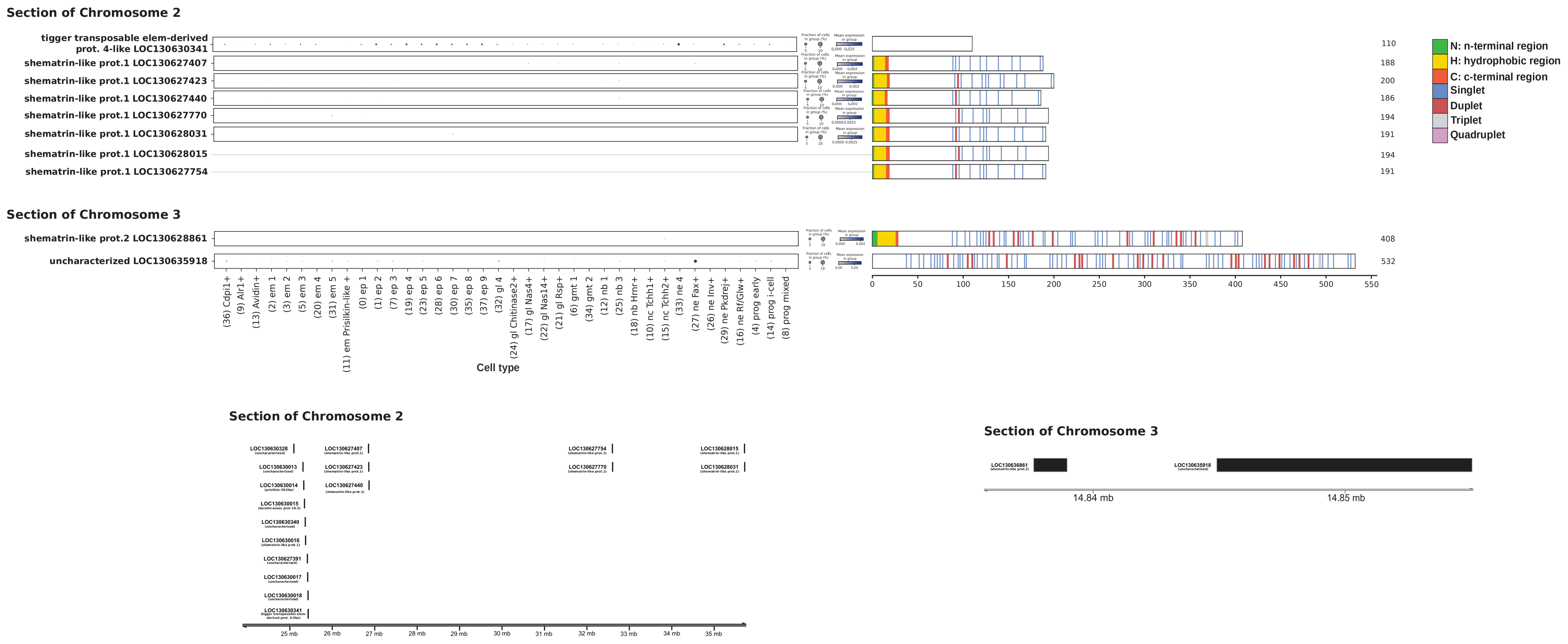

Section of Chromosome 2
tigger transposable elem-derived prot. 4-like LOC130630341
shematrin-like prot.1 LOC130627407
shematrin-like prot.1 LOC130627423
shematrin-like prot.1 LOC130627440
shematrin-like prot.1 LOC130627770
shematrin-like prot.1 LOC130628031
shematrin-like prot.1 LOC130628015
shematrin-like prot.1 LOC130627754
Section of Chromosome 3
shematrin-like prot.2 LOC130628861
uncharacterized LOC130635918
(36) Cdpi1+
(9) Alr1+
(13) Avidin+
(2) em 1
(3) em 2
(5) em 3
(20) em 4
(31) em 5
(11) em Prisilkin-like +
(0) ep 1
(1) ep 2
(7) ep 3
(19) ep 4
(23) ep 5
(28) ep 6
(30) ep 7
(35) ep 8
(37) ep 9
(32) gl 4
(24) gl Chitinase2+
(17) gl Nas4+
(22) gl Nas14+
(21) gl Rsp+
(6) gmt 1
(34) gmt 2
(12) nb 1
(25) nb 3
(18) nb Hmr+
(10) nc Tchh1+
(15) nc Tchh2+
(33) ne 4
(27) ne Fax+
(26) ne Inv+
(29) ne Pkdrej+
(16) ne Rf/Glw+
(4) prog early
(14) prog i-cell
(8) prog mixed
Cell type
Fraction of cells in group (%)
Mean expression in group
110
188
200
186
194
191
194
191
408
532
0
50
100
150
200
250
300
350
400
450
500
550
N: n-terminal region
H: hydrophobic region
C: c-terminal region
Singlet
Duplet
Triplet
Quadruplet
Section of Chromosome 2
LOC130630328 (uncharacterized)
LOC130630013 (uncharacterized)
LOC130630014 (prisilkin-39-like)
LOC130630015 (keratin-assoc prot 19-2)
LOC130630340 (uncharacterized)
LOC130630016 (shematrin-like prot.1)
LOC130627391 (uncharacterized)
LOC130630017 (uncharacterized)
LOC130630018 (uncharacterized)
LOC130630341 (tigger transposable elem-derived prot. 4-like)
LOC130627407 (shematrin-like prot.1)
LOC130627423 (shematrin-like prot.1)
LOC130627440 (shematrin-like prot.1)
LOC130627754 (shematrin-like prot.1)
LOC130627770 (shematrin-like prot.1)
LOC130628015 (shematrin-like prot.1)
LOC130628031 (shematrin-like prot.1)
25 mb
26 mb
27 mb
28 mb
29 mb
30 mb
31 mb
32 mb
33 mb
34 mb
35 mb
Section of Chromosome 3
LOC130636861 (shematrin-like prot.2)
LOC130635918 (uncharacterized)
14.84 mb
14.85 mb

Supplementary References

1. DuBuc TQ*, et al.* Transcription factor AP2 controls cnidarian germ cell induction. *Science* **367**, 757-762 (2020).

2. Chrysostomou E*, et al.* A cellular and molecular analysis of SoxB-driven neurogenesis in a cnidarian. *Elife* **11**, (2022).

3. Varley A, Horkan HR, McMahon ET, Krasovec G, Frank U. Pluripotent, germ cell competent adult stem cells underlie cnidarian regenerative ability and clonal growth. *Curr Biol* **33**, 1883-1892 e1883 (2023).

4. Torok A*, et al.* The cnidarian Hydractinia echinata employs canonical and highly adapted histones to pack its DNA. *Epigenetics Chromatin* **9**, 36 (2016).

5. Nakanishi N, Renfer E, Technau U, Rentzsch F. Nervous systems of the sea anemone Nematostella vectensis are generated by ectoderm and endoderm and shaped by distinct mechanisms. *Development* **139**, 347-357 (2012).

6. Chari T*, et al.* Whole-animal multiplexed single-cell RNA-seq reveals transcriptional shifts across Clytia medusa cell types. *Sci Adv* **7**, eabh1683 (2021).

7. Klompen AML, Sanders SM, Cartwright P. Venom system variation and the division of labor in the colonial hydrozoan Hydractinia symbiolongicarpus. *Toxicon X* **14**, 100113 (2022).

8. Schnitzler CE*, et al.* The genome of the colonial hydroid Hydractinia reveals that their stem cells use a toolkit of evolutionarily shared genes with all animals. *Genome Res* **34**, 498-513 (2024).

9. Surm JM, Landau M, Columbus-Shenkar YY, Moran Y. Sea Anemone Membrane Attack Complex/Perforin Superfamily Demonstrates an Evolutionary Transitional State between Venomous and Developmental Functions. *Mol Biol Evol* **41**, (2024).

10. Mohrlen F, Maniura M, Plickert G, Frohme M, Frank U. Evolution of astacin-like metalloproteases in animals and their function in development. *Evol Dev* **8**, 223-231 (2006).

11. Haridi A. Identification, diversity and domain structure analysis of mucin and mucin-like genes in sea anemone Actinia tenebrosa. *PeerJ* **10**, e13292 (2022).

12. Rubenstein AB*, et al.* Single-cell transcriptional profiles in human skeletal muscle. *Sci Rep* **10**, 229 (2020).

13. Lopez JA, Fain MG, Cadavid LF. The evolution of the immune-type gene family Rhamnospondin in cnidarians. *Gene* **473**, 119-124 (2011).

14. Kong Y*, et al.* Cloning and characterization of Prisilkin-39, a novel matrix protein serving a dual role in the prismatic layer formation from the oyster Pinctada fucata. *J Biol Chem* **284**, 10841-10854 (2009).
