# Supplementary material for: The Hydractinia cell atlas reveals cellular and molecular principles of cnidarian coloniality
Source: Nat Commun. 2025 Mar 3;16:2121. doi: 10.1038/s41467-025-57168-z (PMC11876637; doi:10.1038/s41467-025-57168-z)
Supplement: Supplementary file 3 — Description of Additional Supplementary Files [file 41467_2025_57168_MOESM3_ESM.pdf]

Supplementary Dataset Description

Supplementary Data 1.

Description of treatments per library and sublibrary, and general statistics of mapping when multimapping to multiple loci is and is not allowed. For details on this, check the materials and method section and the code repository.

Supplementary Data 2.

Compiled table of the genes input of the WGCNA and Transcription Factor analysis pipeline. In one tab is the output of diamond blast. Each column corresponds to gene id, protein id, name given by NCBI, accession code of homolog output of diamond blast, sequence coverage of diamond blast, sequence e-value of diamond blast, ortholog match of diamond blast. In the next tab, is the annotation given to the genes input of the WGCNA and Transcription Factor analysis. Each column corresponds to Gene Ontology term product of the eggnog pipeline, Functional categorization product of the eggnog pipeline, WGCNA initial module name, WGCNA renamed module, WGCNA module renamed to be human friendly, Pfam domain name of the transcription factor if found, Pfam superfamily name of the transcription factor if found, reciprocal blast with swissprot transcription factor database, orthofinder symbol of match with transcription factor database, orthofinder family match with transcription factor database, preferred name of the transcription factor if is classified as such, transcription factor class and summary name of the gene id together with transcription factor class.

Supplementary Data 3.

Top 100 marker genes per cluster in resolution 1.5 of the Leiden algorithm calculated through the Wilcoxon rank-sum method. Each column in the corresponding tabs refers to gene names, logarithmic fold changes, p-values, adjusted p-values, scores, ortholog name output of a diamond blast and name given by ncbi.

Supplementary Data 4.

Top 30 marker genes per cluster in resolution 1.5 of the Leiden algorithm calculated through the logarithmic regression method. Each column in the corresponding tabs refers to gene names, scores, ortholog name output of a diamond blast and name given by ncbi.

Supplementary Data 5.

UMAPs corresponding to the genes found in both methods used to calculate defining markers per cluster.

Supplementary Data 6.

Probe sequences used for in situ hybridization.

Supplementary Data 7.

Aggregated cell counts for each broad cell type category and individual cell type, together with the corresponding percentages of these cell counts, hex colour code used per cluster and per broad category, and cell counts per colony part subset.

Supplementary Data 8.

GO biological process terms enrichment (log10(p-value)) per WGCNA module.

Supplementary Data 9.

Multiple sequence alignment of POU domain proteins.

Supplementary Data 10.

Sequence file of all hits for Alr and Alr-like genes in the *Hydractinia symbiolongicarpus* genome.

Supplementary Data 11.

Sequence of vector used for recombinant Alr1 protein expression.

Supplementary Data 12.

Unrooted phylogenetic trees for the four genes associated with biomineralization, Alpha Carbonic Anhydrase, Chitin Synthase, Protocadherin, and Shematrin, obtained with maximum likelihood. In each tree, animal taxa are highlighted in colours with the legend provided accordingly. All sequences belonging to *Hydractinia symbiologicarpus* are highlighted in bold. Leaves on trees include species names, accession numbers if obtained from NCBI, and sequence annotation. Ultrafast bootstrap values are reported on the nodes with red dots if >= 95. For full information on the phylogenetic analyses, the full IQTree outputs are provided for each gene in Supplementary Data 12.

Supplementary Data 13.

IQTree outputs for all Biomineralization gene trees.
