# Supplementary material for: The Hydractinia cell atlas reveals cellular and molecular principles of cnidarian coloniality
Source: Nat Commun. 2025 Mar 3;16:2121. doi: 10.1038/s41467-025-57168-z (PMC11876637; doi:10.1038/s41467-025-57168-z)
Supplement: Supplementary file 9 — Supplementary Data 6 [file 41467_2025_57168_MOESM9_ESM.docx]

**Chitinase2**

DIG-labelled probe covering positions 1069-1278 of the Chitnase2 ORF

**Avidin-like SABER-FISH oligos for probe synthesis**

13.1 GTTTAACTTGGACATCATGTAGATTTTCTGTTTTCTTTTTAATACTCTC

13.2 GCTGGTGCGTTGAGTGTGTTGTTTGCTTTCTGTCCATTTAATACTCTC

13.3 TCACTGCTGTACAATACTTCCCGACCAAATTTCTTCCTTTTTTTAATACTCTC

13.4 GGAAATTTTGTCTTTGCTCGTGTAGGTCACAACCCATTTTAATACTCTC

13.5 GTCAAGGTCCATGTAGTGAGTATGACTTGTTTTCCAGCTTTTTTAATACTCTC

**Prisilkin-39 SABER-FISH oligos for probe synthesis**

30.1 GGTGGATATGGATATGGACACAAAGCATATGGTGACCCTTTAATACTCTC

30.2 TATGGAGGTGTAACTGGCGGATATGGTGGATATGGATTTAATACTCTC

30.3 AAGTGGACATGGAAGTTGATATGGAAGTGGAAGTGGATACGGTTTAATACTCTC

30.4 TTATTCCTGCTTCGTTTGGAACAAAAGTTGACACAGTGAACTTTAATACTCTC

30.5 ACGGTTTGTTGAGCATGAAAGCGATACAAAATTTCTTTGCCTTTAATACTCTC
