# Supplementary material for: The Hydractinia cell atlas reveals cellular and molecular principles of cnidarian coloniality
Source: Nat Commun. 2025 Mar 3;16:2121. doi: 10.1038/s41467-025-57168-z (PMC11876637; doi:10.1038/s41467-025-57168-z)
Supplement: Supplementary file 15 — Supplementary Data 12 [file 41467_2025_57168_MOESM15_ESM.pdf]

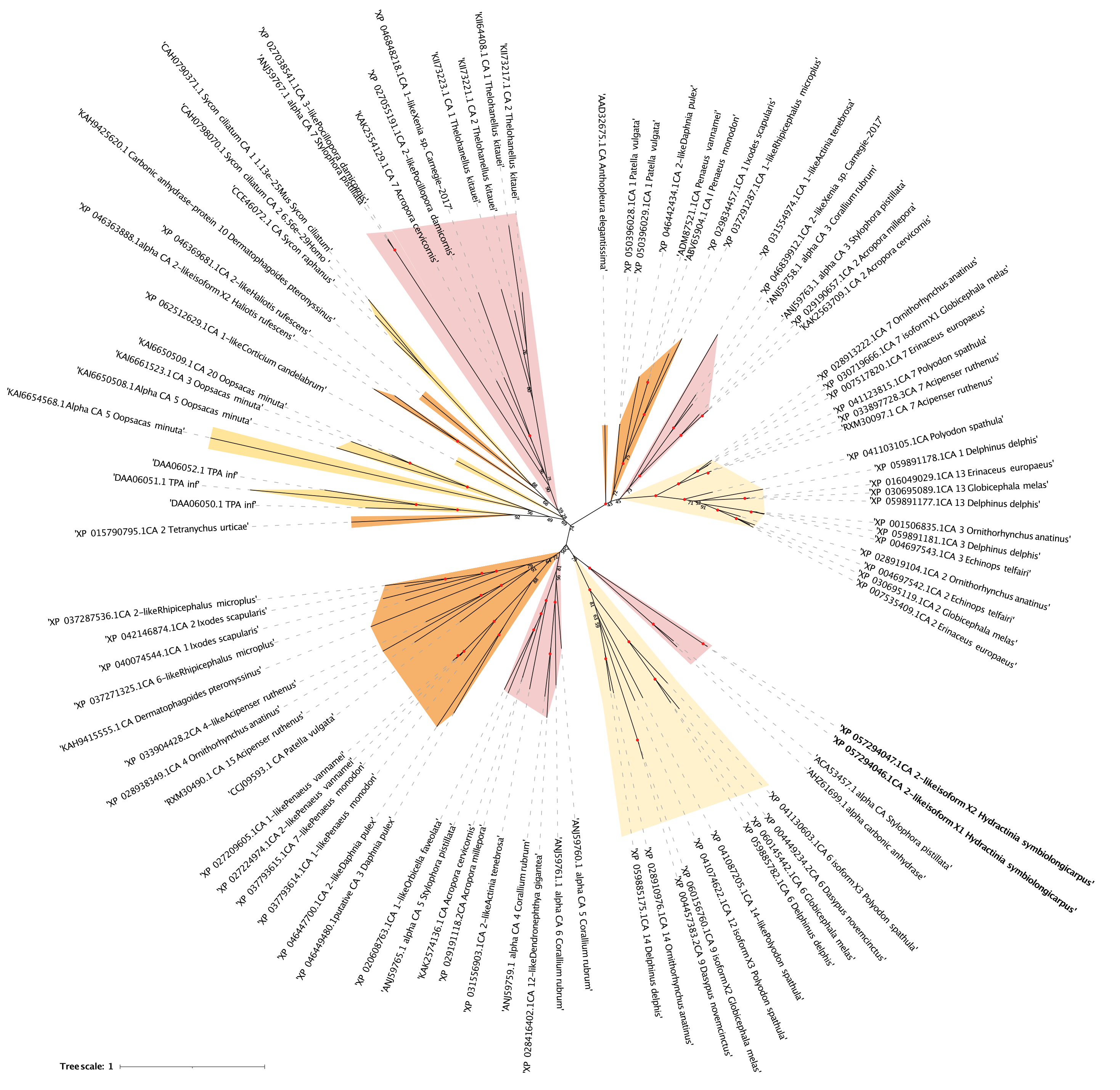

Taxa

- Cnidaria
- Vertebrata
- Porifera
- Bilateria

α- Carbonic Anhydrase

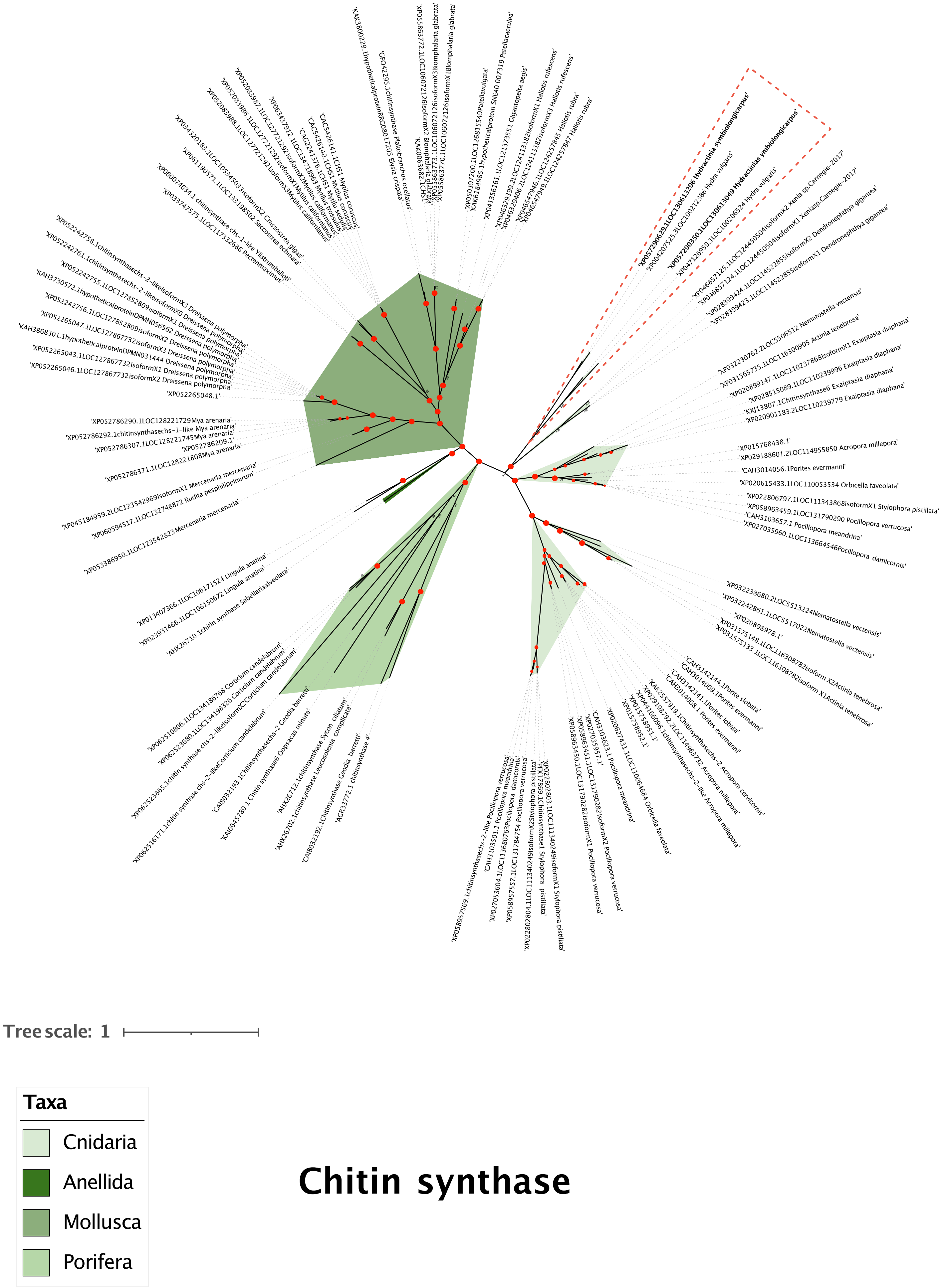

Chitin synthase

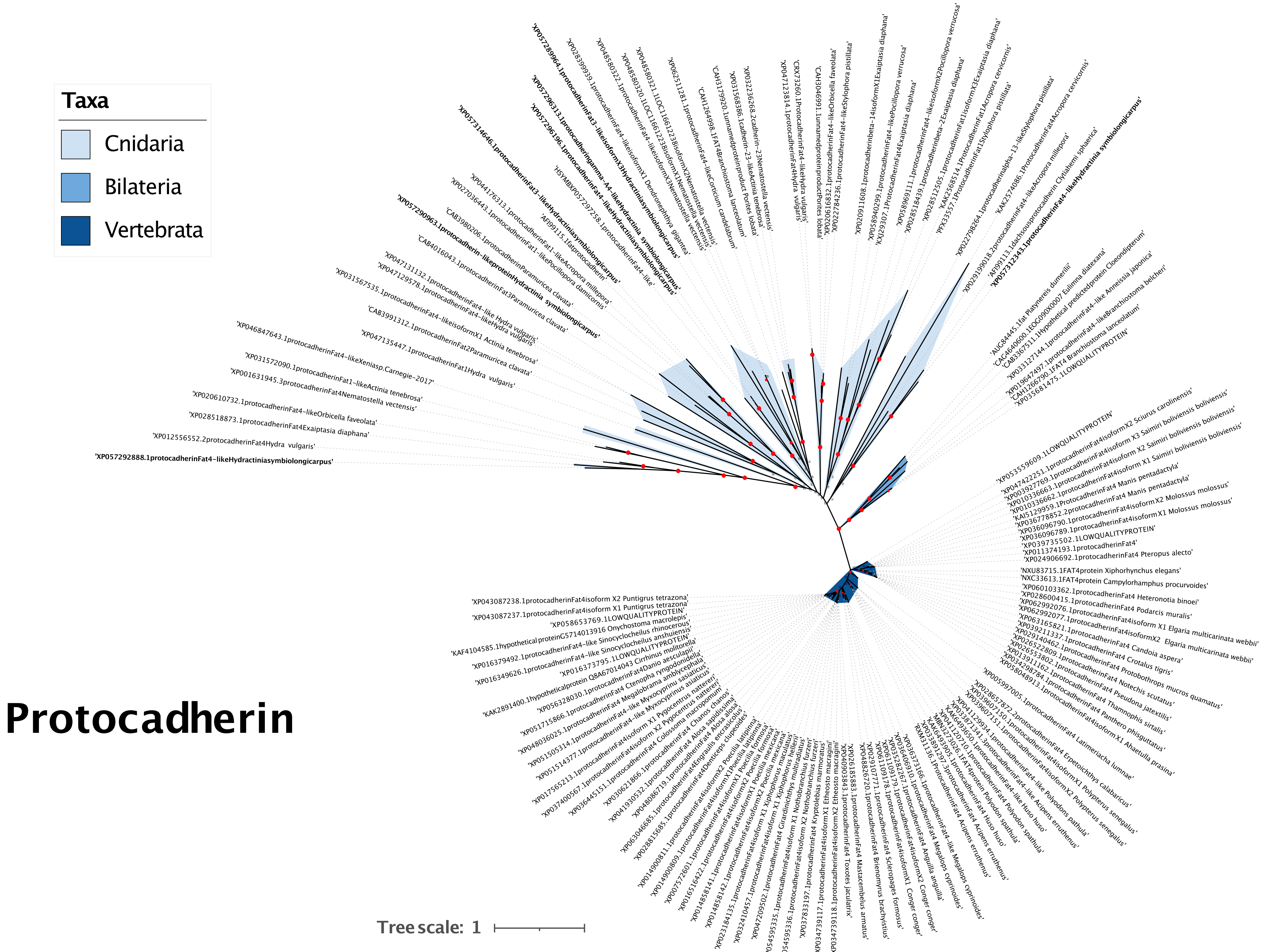
Taxa
Cnidaria
Bilateria
Vertebrata
Protocadherin
Tree scale: 1

Shematrin

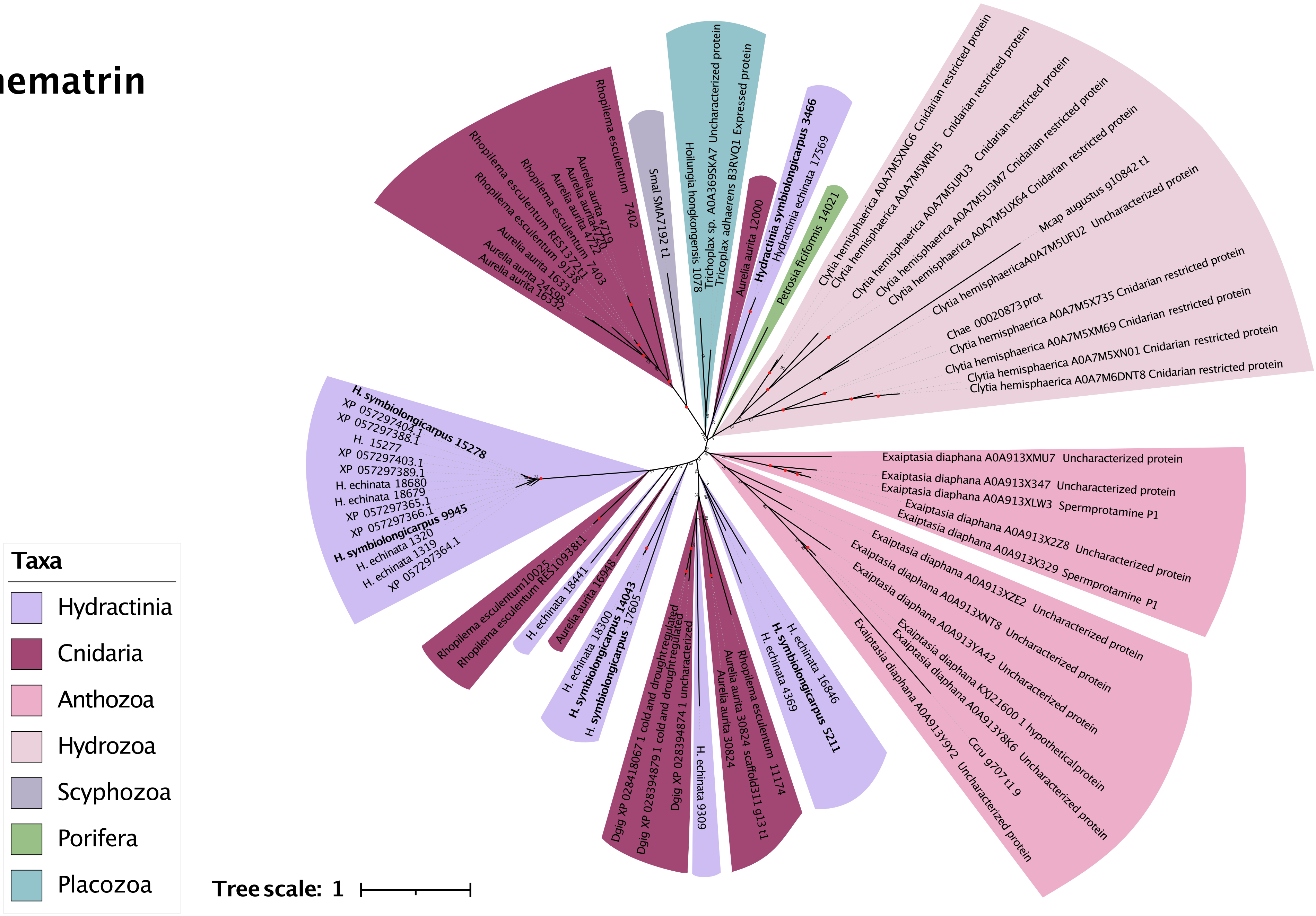

Rhopilema esculentum 7402
Smal SMA7192 t1
Hoilungia hongkongensis 1078
Trichoplax sp. A0A369SKA7 Uncharacterized protein
Tricoplax adhaerens B3RVQ1 Expressed protein
Aurelia aurita 12000
Hydractinia symbiolongicarpus 3466
Hydractinia echinata 17569
Petrosia ficiformis 14021
Clytia hemisphaerica A0A7M5XNG6 Cnidarian restricted protein
Clytia hemisphaerica A0A7M5WRH5 Cnidarian restricted protein
Clytia hemisphaerica A0A7M5UPU3 Cnidarian restricted protein
Clytia hemisphaerica A0A7M5U3M7 Cnidarian restricted protein
Clytia hemisphaerica A0A7M5UX64 Cnidarian restricted protein
Mcap augustus g10842 t1
Clytia hemisphaericaA0A7M5UFU2 Uncharacterized protein
Chae 00020873 prot
Clytia hemisphaerica A0A7M5X735 Cnidarian restricted protein
Clytia hemisphaerica A0A7M5XM69 Cnidarian restricted protein
Clytia hemisphaerica A0A7M5XN01 Cnidarian restricted protein
Clytia hemisphaerica A0A7M6DNT8 Cnidarian restricted protein
Exaiptasia diaphana A0A913XMU7 Uncharacterized protein
Exaiptasia diaphana A0A913X347 Uncharacterized protein
Exaiptasia diaphana A0A913XLW3 Spermprotamine P1
Exaiptasia diaphana A0A913X2Z8 Uncharacterized protein
Exaiptasia diaphana A0A913X329 Spermprotamine P1
Exaiptasia diaphana A0A913XZE2 Uncharacterized protein
Exaiptasia diaphana A0A913XNT8 Uncharacterized protein
Exaiptasia diaphana A0A913YA42 Uncharacterized protein
Exaiptasia diaphana KXJ21600 1 hypotheticalprotein
Exaiptasia diaphana A0A913Y8K6 Uncharacterized protein
Ccru 9707 t1 9
Exaiptasia diaphana A0A913Y9Y2 Uncharacterized protein
H. echinata 16846
H. symbiolongicarpus 5211
H. echinata 4369
Rhopilema esculentum 11174
Aurelia aurita 30824 scaffold311 g13 t1
Aurelia aurita 30824
H. echinata 9309
Dgig XP 028394874 1 uncharacterized
Dgig XP 028394879 1 cold and drought regulated
Dgig XP 028418067 1 cold and drought regulated
H. echinata 18300
H. symbiolongicarpus 14043
H. symbiolongicarpus 17605
Aurelia aurita 16948
H. echinata 18441
Rhopilema esculentum10025
Rhopilema esculentum RES10938t1
H. symbiolongicarpus 15278
XP 057297404.1
XP 057297388.1
H. 15277
XP 057297403.1
XP 057297389.1
H. echinata 18680
H. echinata 18679
XP 057297365.1
XP 057297366.1
H. symbiolongicarpus 9945
H. echinata 1320
H. echinata 1319
XP 057297364.1
Rhopilema esculentum 7403
Rhopilema esculentum RES13721t1
Rhopilema esculentum 9138
Aurelia aurita 4719
Aurelia aurita 4720
Aurelia aurita 4722
Aurelia aurita 16331
Aurelia aurita 24598
Aurelia aurita 16332
Taxa
Hydractinia
Cnidaria
Anthozoa
Hydrozoa
Scyphozoa
Porifera
Placozoa
Tree scale: 1
