# Supplementary figures and images for: The Hydractinia cell atlas reveals cellular and molecular principles of cnidarian coloniality
Source: Nat Commun. 2025 Mar 3;16:2121. doi: 10.1038/s41467-025-57168-z (PMC11876637; doi:10.1038/s41467-025-57168-z)

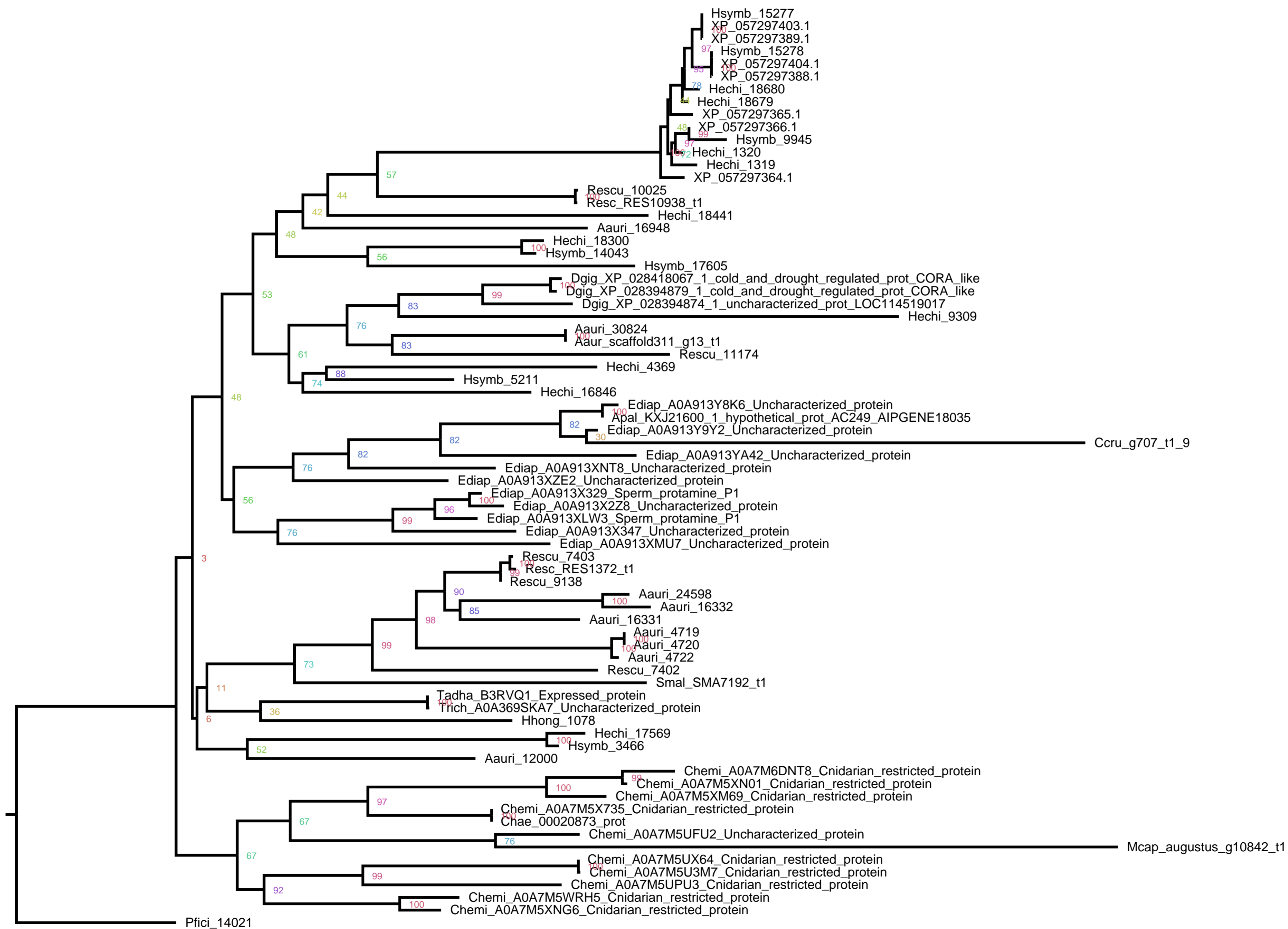

0.5

Supplement: Supplementary file 16 — Supplementary Data 13 [file 41467_2025_57168_MOESM16_ESM.zip › IQ_Tree_output/shematrin/shematrin_prot_cni_10_MAFFT_trim.fasta.treefile.pdf]

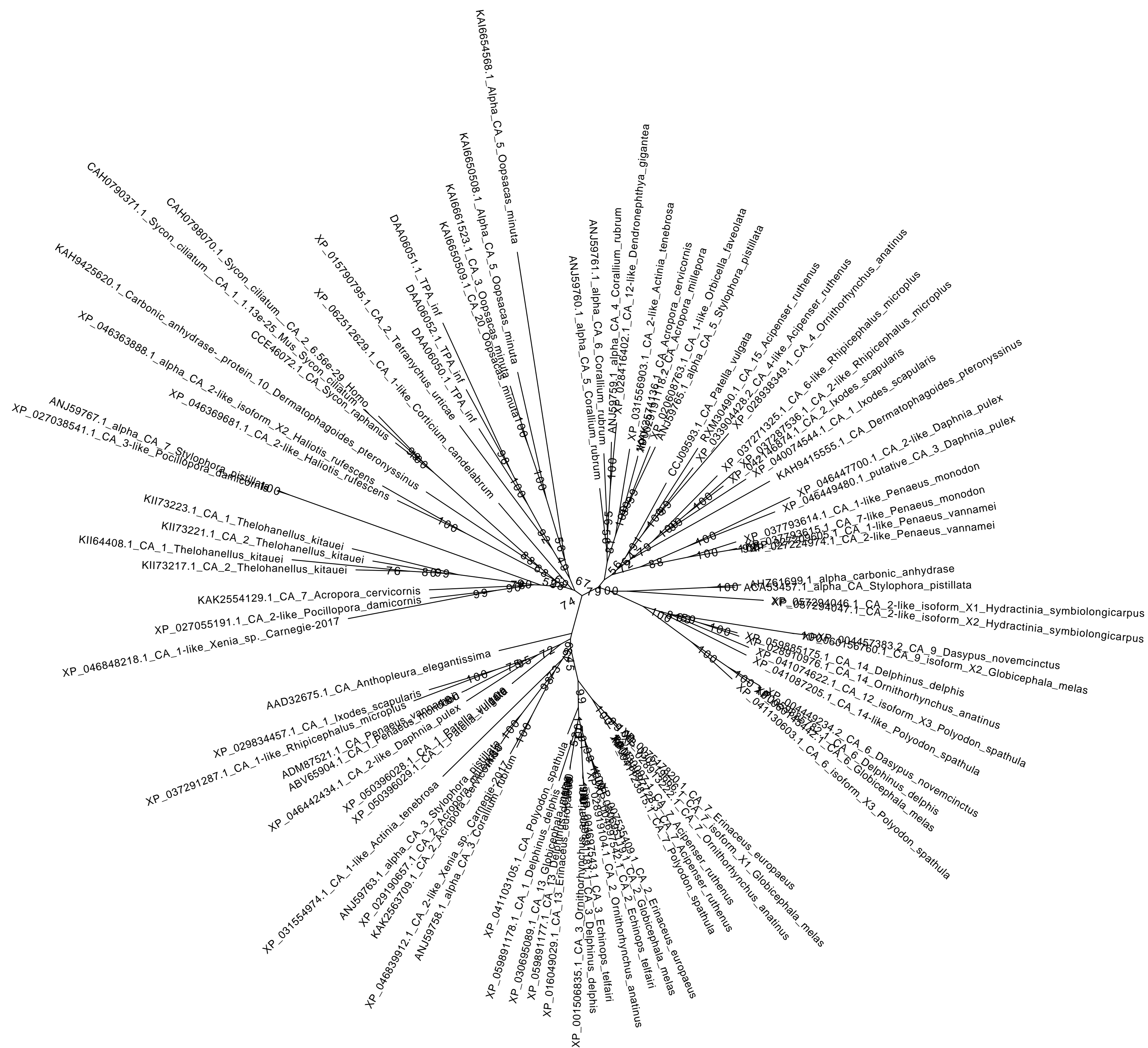

Supplement: Supplementary file 16 — Supplementary Data 13 [file 41467_2025_57168_MOESM16_ESM.zip › IQ_Tree_output/Alpha_carbonic_Anhydrase/Edited_tree/All_alpha_carbonic_LBAremoved_NoBAC.fasta.linsi.trim.tre.pdf]

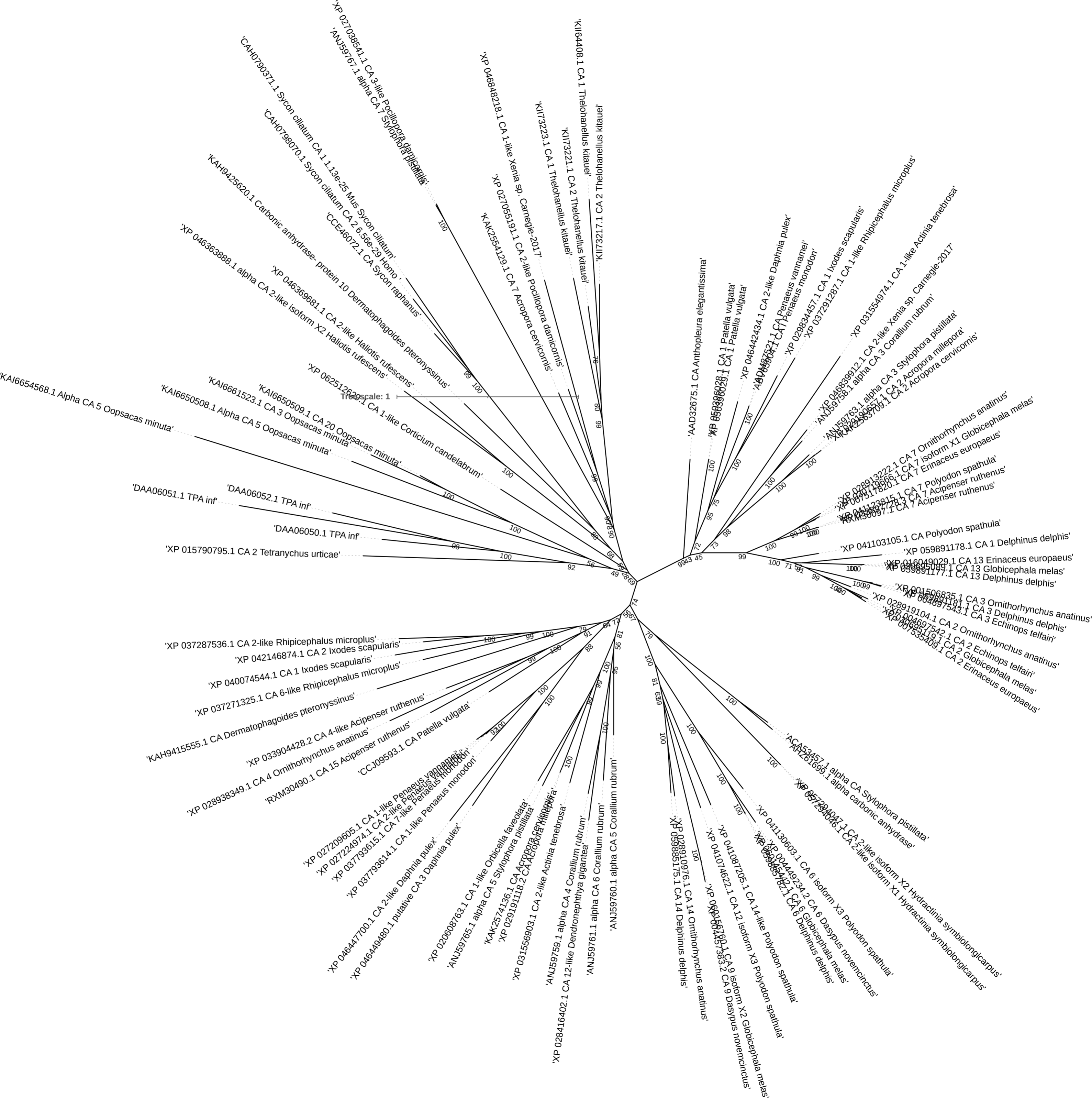

Supplement: Supplementary file 16 — Supplementary Data 13 [file 41467_2025_57168_MOESM16_ESM.zip › IQ_Tree_output/Alpha_carbonic_Anhydrase/Edited_tree/Alpha_carbonic_anhydrase.pdf]

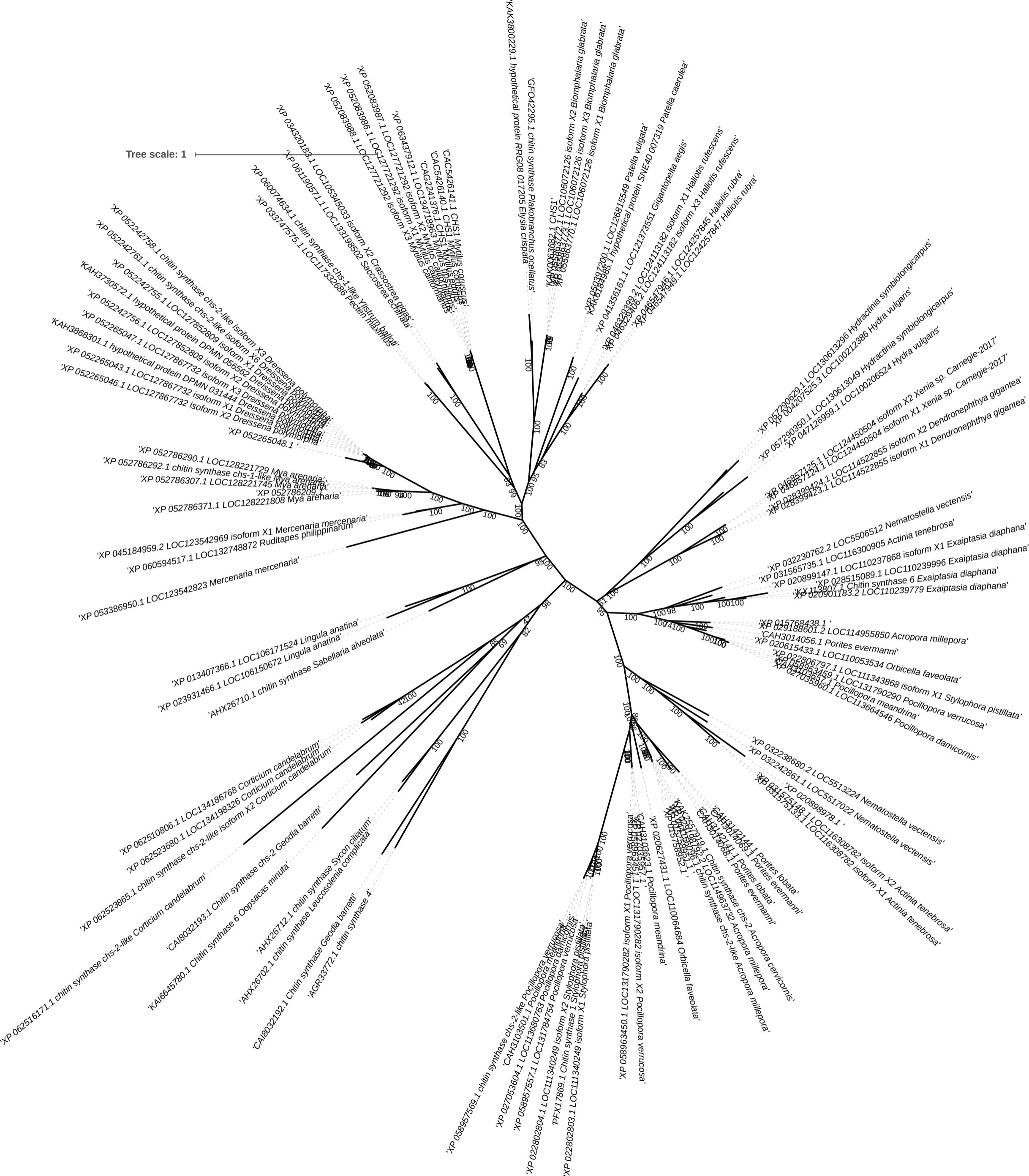

Supplement: Supplementary file 16 — Supplementary Data 13 [file 41467_2025_57168_MOESM16_ESM.zip › IQ_Tree_output/Chitin_synthase/Edited_tree/Chitin_edited.pdf]
