# Supplementary material for: The Hydractinia cell atlas reveals cellular and molecular principles of cnidarian coloniality
Source: Nat Commun. 2025 Mar 3;16:2121. doi: 10.1038/s41467-025-57168-z (PMC11876637; doi:10.1038/s41467-025-57168-z)
Supplement: Supplementary file 17 — Reporting Summary [file 41467_2025_57168_MOESM17_ESM.pdf]

nature portfolio

Corresponding author(s): Helen R Horkan, Uri Frank, Jordi Solana.

Last updated by author(s): Dec 27, 2024

Reporting Summary

Nature Portfolio wishes to improve the reproducibility of the work that we publish. This form provides structure for consistency and transparency in reporting. For further information on Nature Portfolio policies, see our Editorial Policies and the Editorial Policy Checklist.

Statistics

For all statistical analyses, confirm that the following items are present in the figure legend, table legend, main text, or Methods section.

n/a	Confirmed	
☐	☒	The exact sample size (*n*) for each experimental group/condition, given as a discrete number and unit of measurement
☐	☒	A statement on whether measurements were taken from distinct samples or whether the same sample was measured repeatedly
☐	☒	The statistical test(s) used AND whether they are one- or two-sided<br>*Only common tests should be described solely by name; describe more complex techniques in the Methods section.*
☒	☐	A description of all covariates tested
☐	☒	A description of any assumptions or corrections, such as tests of normality and adjustment for multiple comparisons
☐	☒	A full description of the statistical parameters including central tendency (e.g. means) or other basic estimates (e.g. regression coefficient) AND variation (e.g. standard deviation) or associated estimates of uncertainty (e.g. confidence intervals)
☐	☒	For null hypothesis testing, the test statistic (e.g. *F*, *t*, *r*) with confidence intervals, effect sizes, degrees of freedom and *P* value noted<br>*Give P values as exact values whenever suitable.*
☐	☒	For Bayesian analysis, information on the choice of priors and Markov chain Monte Carlo settings
☒	☐	For hierarchical and complex designs, identification of the appropriate level for tests and full reporting of outcomes
☐	☒	Estimates of effect sizes (e.g. Cohen's *d*, Pearson's *r*), indicating how they were calculated

Our web collection on statistics for biologists contains articles on many of the points above.

Software and code

Policy information about availability of computer code

Data collection	No software was used for data collection.
Data analysis	Code:<br>Diamond v2.0.8.146, EggNOG Mapper v2, FastQC v0.11.9, CutAdapt v2.8, Pairfq v0.17, Dropseq_tools-2.3.0, STAR-2.3.7a, SPLiTseq toolbox v1.0, Picard v2.21.1, MUON v0.1.5, Harmony v0.1, Scanpy v1.9.3, scCODA v0.1.9, DESeq2 v1.30.0, InterProScan, BLAST+ v2.10.0, OrthoFinder v2.3.8, Python v3.8.10, R v4.0.3, ComplexHeatmap v2.II.I, ggplot2 v.3.3.3, igraph v0.11.6, Kamada-Kawai, mclust v6.11, topGo v2.52.0, SignalP 6.0, OligoMiner v1.0, IQTree 2 v.2.2.0.3, MrBayes 3.2.7a, Clustal Omega v1.2.2, samtools v1.10, Fiji v2.9.0.

For manuscripts utilizing custom algorithms or software that are central to the research but not yet described in published literature, software must be made available to editors and reviewers. We strongly encourage code deposition in a community repository (e.g. GitHub). See the Nature Portfolio guidelines for submitting code & software for further information.

Data

Policy information about availability of data

All manuscripts must include a data availability statement. This statement should provide the following information, where applicable:

- Accession codes, unique identifiers, or web links for publicly available datasets
- A description of any restrictions on data availability
- For clinical datasets or third party data, please ensure that the statement adheres to our policy

The datasets supporting the conclusions of this article are available in:
GEO (scRNA-seq reads): GSE269914
The processed annotated dataset was uploaded into the UCSC cell browser in:
http://cells.ucsc.edu/?ds=hydractinia

BUSCO database https://busco.ezlab.org/
nr database https://www.ncbi.nlm.nih.gov/refseq/about/nonredundantproteins/
Pfam database http://pfam-legacy.xfam.org/
PANTHER database https://pantherdb.org
SUPERFAMILY database https://superfam.org/SUPERFAMILY/
EggNOG database http://eggnog5.embl.de

Research involving human participants, their data, or biological material

Policy information about studies with human participants or human data. See also policy information about sex, gender (identity/presentation), and sexual orientation and race, ethnicity and racism.

Reporting on sex and gender	This study did not involve research in humans.
Reporting on race, ethnicity, or other socially relevant groupings	This study did not involve research in humans.
Population characteristics	This study did not involve research in humans.
Recruitment	This study did not involve research in humans.
Ethics oversight	This study did not involve research in humans.

Note that full information on the approval of the study protocol must also be provided in the manuscript.

Field-specific reporting

Please select the one below that is the best fit for your research. If you are not sure, read the appropriate sections before making your selection.

[x] Life sciences [ ] Behavioural & social sciences [ ] Ecological, evolutionary & environmental sciences

For a reference copy of the document with all sections, see nature.com/documents/nr-reporting-summary-flat.pdf

Life sciences study design

All studies must disclose on these points even when the disclosure is negative.

Sample size	No statistical method was used to determine sample size. The sample size for each sub-library was determined by performing pilot studies to assess the number of cells obtained from the various tissue types using both microscopy and flow cytometry. The minimum number of cells required for a single sample was determined in Garcia-Castro et al 2021.
Data exclusions	Cells which fell below a minimum threshold of 50 genes per cell were excluded from the dataset to ensure robust identification of live cells.
Replication	Out dataset includes 2 biological replicates for each sample type. All replicates were successful.
Randomization	This study does not require randomization as no effect is being tested, rather it is an assessment of the cell type composition of homeostatic animals.
Blinding	Blinding is not relevant for this study.

Reporting for specific materials, systems and methods

We require information from authors about some types of materials, experimental systems and methods used in many studies. Here, indicate whether each material, system or method listed is relevant to your study. If you are not sure if a list item applies to your research, read the appropriate section before selecting a response.

Materials & experimental systems

n/a	Involved in the study
☐	☒ Antibodies
☒	☐ Eukaryotic cell lines
☒	☐ Palaeontology and archaeology
☐	☒ Animals and other organisms
☒	☐ Clinical data
☒	☐ Dual use research of concern
☒	☐ Plants

Methods

n/a	Involved in the study
☒	☐ ChIP-seq
☒	☐ Flow cytometry
☒	☐ MRI-based neuroimaging

Antibodies

Antibodies used	Custom Alr1 antibody generated in this study. Secondary Alexa Fluor 594 goat anti-mouse Invitrogen Catalog # A-11005.
Validation	Primary Alr1 antibody was validated using western blot. Secondary antibody commercially validated.

Animals and other research organisms

Policy information about studies involving animals; ARRIVE guidelines recommended for reporting animal research, and Sex and Gender in Research

Laboratory animals	All samples were taken from Hydractinia symbiolongicarpus male clone 291-10, a common lab strain. Culture conditions are described in the manuscript.
Wild animals	This study did not involve wild animals.
Reporting on sex	This study sampled only male clone 291-10.
Field-collected samples	This study did not include field collected samples.
Ethics oversight	No ethical approval is required for use of Cnidarians.

Note that full information on the approval of the study protocol must also be provided in the manuscript.

Plants

Seed stocks	*Report on the source of all seed stocks or other plant material used. If applicable, state the seed stock centre and catalogue number. If plant specimens were collected from the field, describe the collection location, date and sampling procedures.*
Novel plant genotypes	*Describe the methods by which all novel plant genotypes were produced. This includes those generated by transgenic approaches, gene editing, chemical/radiation-based mutagenesis and hybridization. For transgenic lines, describe the transformation method, the number of independent lines analyzed and the generation upon which experiments were performed. For gene-edited lines, describe the editor used, the endogenous sequence targeted for editing, the targeting guide RNA sequence (if applicable) and how the editor was applied.*
Authentication	*Describe any authentication procedures for each seed stock used or novel genotype generated. Describe any experiments used to assess the effect of a mutation and, where applicable, how potential secondary effects (e.g. second site T-DNA insertions, mosiacism, off-target gene editing) were examined.*
