# Supplementary material for: The Hydractinia cell atlas reveals cellular and molecular principles of cnidarian coloniality
Source: Nat Commun. 2025 Mar 3;16:2121. doi: 10.1038/s41467-025-57168-z (PMC11876637; doi:10.1038/s41467-025-57168-z)

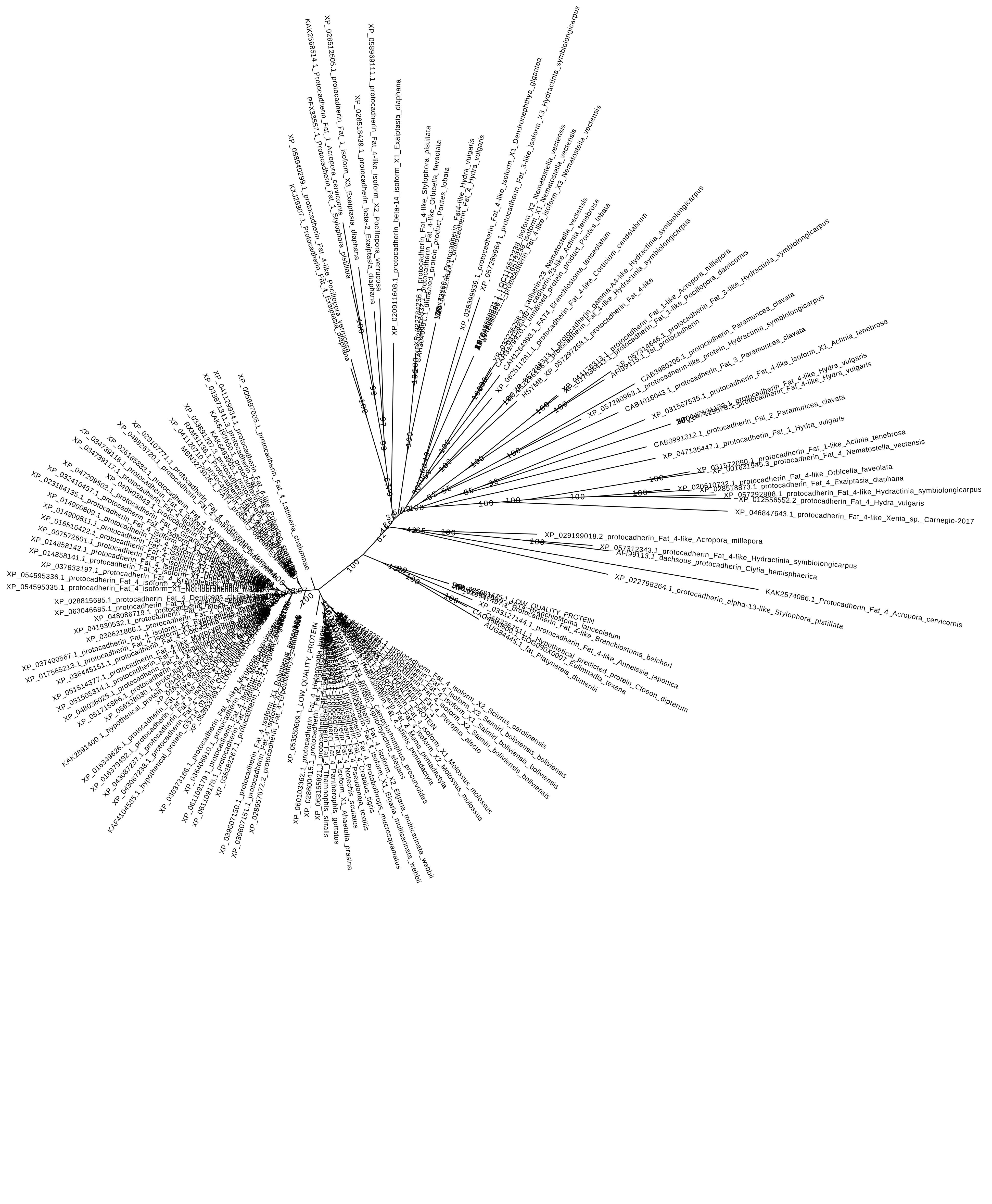

Supplement: Supplementary file 16 — Supplementary Data 13 [file Protochaderin_all.fa.clean.linsi.trim.tre.pdf › IQ_Tree_output/Protochaderin/Protochaderin_all.fa.clean.linsi.trim.tre.pdf]
