# Supplementary material for: The Hydractinia cell atlas reveals cellular and molecular principles of cnidarian coloniality
Source: Nat Commun. 2025 Mar 3;16:2121. doi: 10.1038/s41467-025-57168-z (PMC11876637; doi:10.1038/s41467-025-57168-z)
Supplement: Supplementary file 16 — Supplementary Data 13 [file Chitin_syntase.fa.clean.linsi.trim.tre.pdf › IQ_Tree_output/Chitin_synthase/Edited_tree/Chitin_syntase.fa.clean.linsi.trim.tre.pdf]

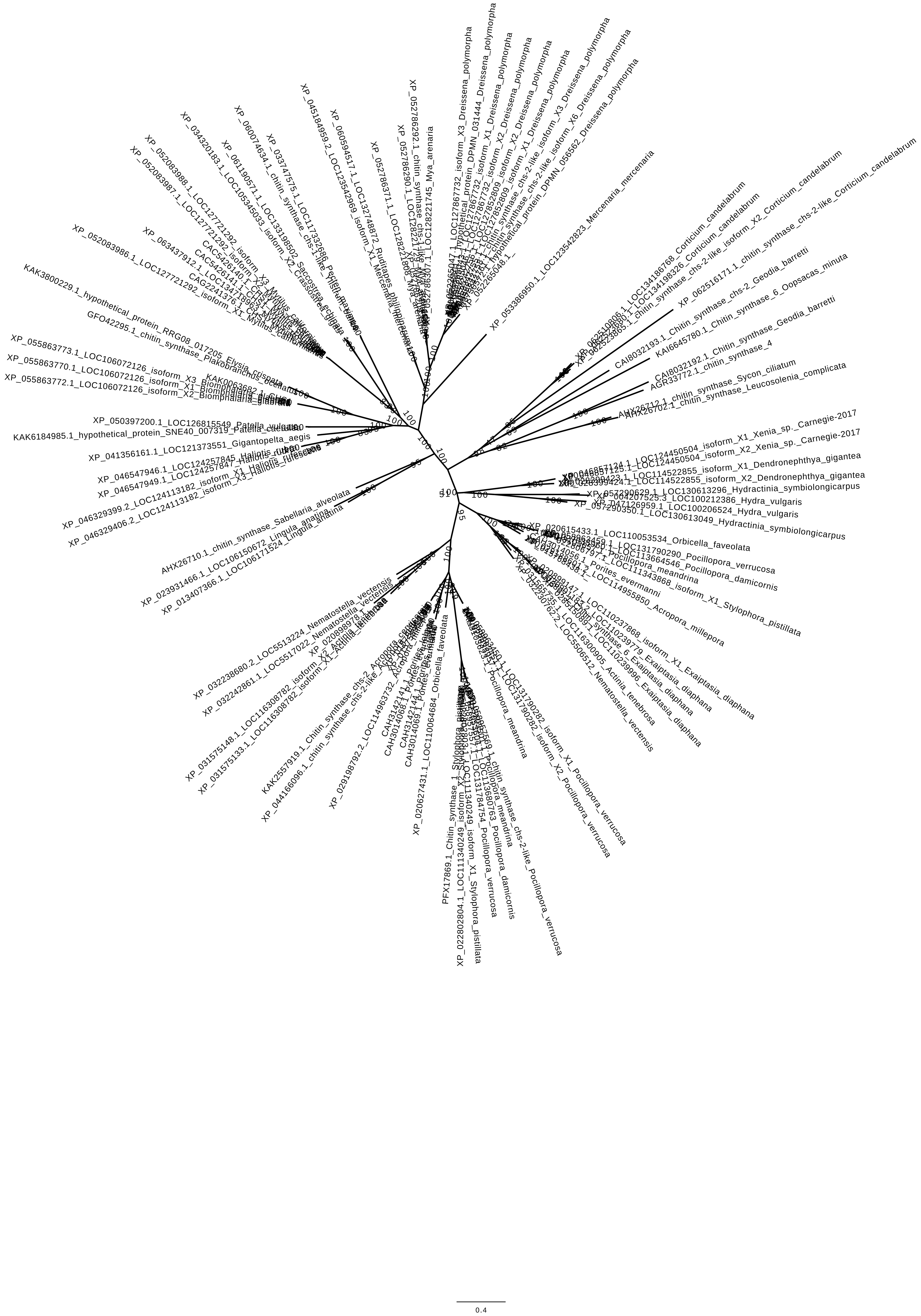

XP_053380950.1_LOC123542823_Mercenaria_mercenaria
XP_062516171.1_chitin_synthase_chs-2-like_isoform_X2_Corticium_candelabrum
XP_062516171.1_chitin_synthase_chs-2-like_Corticium_candelabrum
XP_062516171.1_chitin_synthase_chs-2_Geodia_barretti
CAI8032193.1_Chitin_synthase_chs-2_Geodia_barretti
KAI6645780.1_Chitin_synthase_6_Oopsacas_minuta
CAI8032192.1_Chitin_synthase_Geodia_barretti
AGR33772.1_chitin_synthase_4
AHX26712.1_chitin_synthase_Sycon_ciliatum
AHX26702.1_chitin_synthase_Leucosolenia_complicata
XP_034320183.1_LOC117577324_isoform_X1_Xenia_sp._Carnegie-2017
XP_034320183.1_LOC124450504_isoform_X2_Xenia_sp._Carnegie-2017
XP_046857124.1_LOC114522855_isoform_X1_Dendronephthya_gigantea
XP_028399423.1_LOC114522855_isoform_X2_Dendronephthya_gigantea
XP_057302629.1_LOC130613296_Hydractinia_symbiolongicarpus
XP_047126959.1_LOC100212386_Hydra_vulgaris
XP_037290350.1_LOC100206524_Hydra_vulgaris
XP_057302573.1_LOC130613049_Hydractinia_symbiolongicarpus
XP_020615433.1_LOC110053534_Orbicella_faveolata
XP_058948459.1_LOC131790290_Pocillopora_verrucosa
XP_058948459.1_LOC113664546_Pocillopora_damicornis
XP_022802804.1_LOC111343868_isoform_X1_Stylophora_pistillata
KAK3800229.1_hypothetical_protein_RRG08_017205_Elysia_crispata
GFO42295.1_chitin_synthase_Plakobranchus_ocellatus
XP_055863773.1_LOC106072126_isoform_X3_Biomphalaria_glabrata
XP_055863770.1_LOC106072126_isoform_X1_Biomphalaria_glabrata
XP_055863772.1_LOC106072126_isoform_X2_Biomphalaria_glabrata
XP_050397200.1_LOC126815549_Patella_vulgata
KAK6184985.1_hypothetical_protein_SNE40_007319_Patella_caerulea
XP_041356161.1_LOC121373551_Gigantopelta_aegis
XP_046547946.1_LOC124257845_Haliotis_rubra
XP_046547949.1_LOC124257847_Haliotis_rufescens
XP_046329399.2_LOC124113182_isoform_X1_Haliotis_rufescens
XP_046329406.2_LOC124113182_isoform_X3_Haliotis_rufescens
AHX26710.1_chitin_synthase_Sabellaria_alveolata
XP_023931466.1_LOC106150672_Lingula_anatina
XP_013407366.1_LOC106171524_Lingula_anatina
XP_032238680.2_LOC5513224_Nematostella_vectensis
XP_032242861.1_LOC5517022_Nematostella_vectensis
XP_031575148.1_LOC116308782_isoform_X1_Actinia_tenebrosa
XP_031575133.1_LOC116308782_isoform_X2_Actinia_tenebrosa
KAK2557919.1_Chitin_synthase_chs-2_Acropora_cervicornis
XP_044166096.1_chitin_synthase_chs-2-like_Acropora_millepora
XP_029198792.2_LOC114963732_Acropora_millepora
XP_020627431.1_LOC110064684_Orbicella_faveolata
PFX17869.1_Chitin_synthase_1_Stylophora_pistillata
XP_022802804.1_LOC111340249_isoform_X1_Stylophora_pistillata
XP_052783371.1_LOC128221745_Mya_arenaria
XP_045184959.2_LOC123542969_isoform_X1_Mercenaria_mercenaria
XP_052083986.1_LOC127721292_isoform_X1_Mytilus_californianus
XP_052083987.1_LOC127721292_isoform_X2_Mytilus_californianus
XP_052083988.1_LOC127721292_isoform_X3_Mytilus_californianus
0.4
