# Supplementary material for: The Hydractinia cell atlas reveals cellular and molecular principles of cnidarian coloniality
Source: Nat Commun. 2025 Mar 3;16:2121. doi: 10.1038/s41467-025-57168-z (PMC11876637; doi:10.1038/s41467-025-57168-z)
Supplement: Supplementary file 16 — Supplementary Data 13 [file Chitin_syntase.pdf › IQ_Tree_output/Chitin_synthase/Edited_tree/Chitin_syntase.pdf]

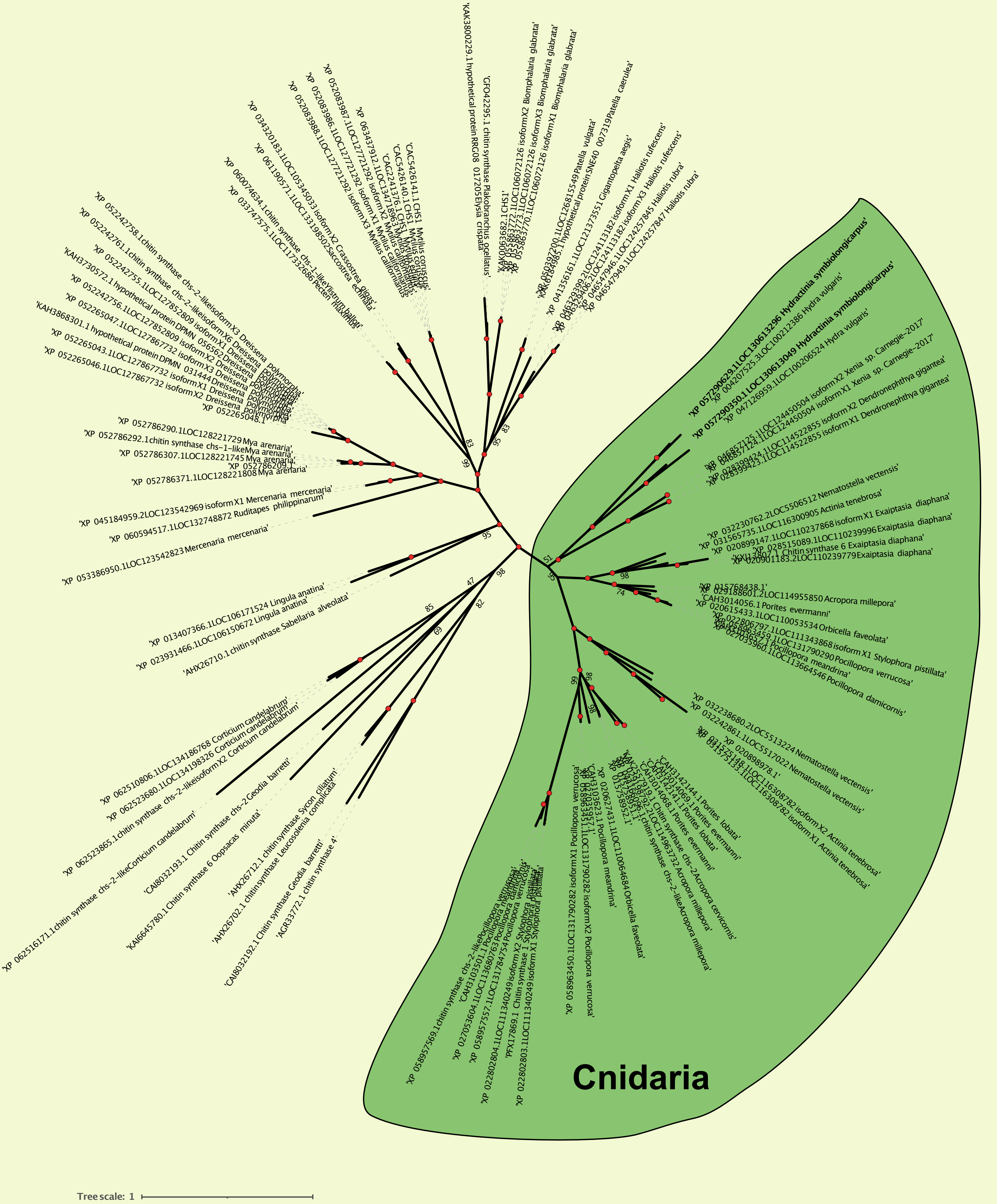
Cnidaria
Tree scale: 1
'XP 057290629.1LOC130613296 Hydractinia symbiolongicarpus'
'XP 057290350.1LOC130613049 Hydractinia symbiolongicarpus'
'XP 004207525.3LOC100212386 Hydra vulgaris'
'XP 047126959.1LOC100206524 Hydra vulgaris'
'XP 046857125.1LOC124450504 isoformX2 Xenia sp. Carnegie-2017'
'XP 046857124.1LOC124450504 isoformX1 Xenia sp. Carnegie-2017'
'XP 028399424.1LOC114522855 isoformX2 Dendronephthya gigantea'
'XP 028399423.1LOC114522855 isoformX1 Dendronephthya gigantea'
'XP 032230762.2LOC5506512 Nematostella vectensis'
'XP 031565735.1LOC116300905 Actinia tenebrosa'
'XP 020899147.1LOC110237868 isoformX1 Exaiptasia diaphana'
'XP 028515089.1LOC110239996 Exaiptasia diaphana'
'KXJ13807.1 Chitin synthase 6 Exaiptasia diaphana'
'XP 020901183.2LOC110239779 Exaiptasia diaphana'
'XP 015768438.1'
'XP 029188601.2LOC114955850 Acropora millepora'
'CAH3014056.1 Porites evermanni'
'XP 020615433.1LOC110053534 Orbicella faveolata'
'XP 022806797.1LOC111343868 isoformX1 Stylophora pistillata'
'XP 058963459.1LOC131790290 Pocillopora verrucosa'
'XP 027035960.1LOC113664546 Pocillopora damicornis'
'XP 032238680.2LOC5513224 Nematostella vectensis'
'XP 032242861.1LOC5517022 Nematostella vectensis'
'XP 020898978.1'
'XP 031575148.1LOC116308782 isoformX2 Actinia tenebrosa'
'XP 031575135.1LOC116308782 isoformX1 Actinia tenebrosa'
'CAH3142144.1 Porites lobata'
'CAH3014069.1 Porites evermanni'
'CAH3142141.1 Porites lobata'
'CAH3014068.1 Porites evermanni'
'XP 020627431.1LOC110064684 Orbicella faveolata'
'XP 058963450.1LOC131790282 isoformX2 Pocillopora verrucosa'
'XP 058963450.1LOC131790282 isoformX1 Pocillopora verrucosa'
'XP 058957569.1chitin synthase chs-2-likePocillopora verrucosa'
'CAH3103501.1 Pocillopora meandrina'
'XP 027053604.1LOC113680763 Pocillopora damicornis'
'XP 058957557.1LOC131784754 Pocillopora verrucosa'
'XP 022802804.1LOC111340249 isoformX2 Stylophora pistillata'
'PFX17869.1 Chitin synthase 1 Stylophora pistillata'
'XP 022802803.1LOC111340249 isoformX1 Stylophora pistillata'
'KAK3800229.1 hypothetical protein RRG08 017205Elysia crispata'
'GFO42295.1 chitin synthase Plakobranchus ocellatus'
'KAK0063682.1CHS1'
'XP 055863772.1LOC106072126 isoformX2 Biomphalaria glabrata'
'XP 055863773.1LOC106072126 isoformX3 Biomphalaria glabrata'
'XP 055863770.1LOC106072126 isoformX1 Biomphalaria glabrata'
'XP 050397200.1LOC126815549 Patella vulgata'
'KAK6184985.1 hypothetical protein SNE40 007319 Patella caerulea'
'XP 041356161.1LOC121373551 Gigantopelta aegis'
'XP 046547946.1LOC124257845 Haliotis rubra'
'XP 046547949.1LOC124257847 Haliotis rubra'
'CAC54261.1 CHS1 Mytilus coruscus'
'CAC54261.40.1 CHS1 Mytilus edulis'
'XP 063437912.1LOC127721292 isoformX2 Mytilus californianus'
'XP 052083987.1LOC127721292 isoformX1 Mytilus californianus'
'XP 052083986.1LOC127721292 isoformX3 Mytilus californianus'
'XP 052083988.1LOC127721292 isoformX2 Crassostrea gigas'
'XP 034320183.1LOC105345033 isoformX2 Crassostrea gigas'
'XP 061190571.1LOC133198502Saccostrea echinata'
'XP 060074634.1chitin synthase chs-1-likeYlistrum balloti'
'XP 033747575.1LOC117332686 Pecten maximus'
'XP 052242758.1chitin synthase chs-2-likeisoformX3 Dreissena polymorpha'
'XP 052242761.1chitin synthase chs-2-likeisoformX6 Dreissena polymorpha'
'KAH3730572.1 hypothetical protein DPMN 056562 Dreissena polymorpha'
'XP 052242755.1LOC127852809 isoformX1 Dreissena polymorpha'
'XP 052242756.1LOC127852809 isoformX2 Dreissena polymorpha'
'XP 052265047.1LOC127867732 isoformX3 Dreissena polymorpha'
'KAH3868301.1 hypothetical protein DPMN 031444 Dreissena polymorpha'
'XP 052265043.1LOC127867732 isoformX1 Dreissena polymorpha'
'XP 052265046.1LOC127867732 isoformX2 Dreissena polymorpha'
'XP 052265048.1'
'XP 052786290.1LOC128221729 Mya arenaria'
'XP 052786292.1chitin synthase chs-1-likeMya arenaria'
'XP 052786307.1LOC128221745 Mya arenaria'
'XP 052786209.1'
'XP 052786371.1LOC128221808 Mya arenaria'
'XP 045184959.2LOC123542969 isoformX1 Mercenaria mercenaria'
'XP 060594517.1LOC132748872 Ruditapes philippinarum'
'XP 053386950.1LOC123542823 Mercenaria mercenaria'
'XP 013407366.1LOC106171524 Lingula anatina'
'XP 023931466.1LOC106150672 Lingula anatina'
'AHX26710.1 chitin synthase Sabellaria alveolata'
'XP 062510806.1LOC134186768 Corticium candelabrum'
'XP 062523680.1LOC134198326 Corticium candelabrum'
'XP 062523865.1chitin synthase chs-2-likeisoformX2 Corticium candelabrum'
'XP 062516171.1chitin synthase chs-2-likeCorticium candelabrum'
'CAI8032193.1 Chitin synthase chs-2Geodia barretti'
'KAI6645780.1 Chitin synthase 6 Oopsacas minuta'
'AHX26712.1 chitin synthase Sycon ciliatum'
'AHX26702.1 chitin synthase Leucosolenia complicata'
'CAI8032192.1 Chitin synthase Geodia barretti'
'AGR33772.1 chitin synthase 4'
83
99
95
83
95
51
95
98
74
47
98
85
82
69
99
98
98
